# Prolonged SARS-CoV-2 RNA virus shedding and lymphopenia are hallmarks of COVID-19 in cancer patients with poor prognosis

**DOI:** 10.1038/s41418-021-00817-9

**Published:** 2021-07-06

**Authors:** Anne-Gaëlle Goubet, Agathe Dubuisson, Arthur Geraud, François-Xavier Danlos, Safae Terrisse, Carolina Alves Costa Silva, Damien Drubay, Lea Touri, Marion Picard, Marine Mazzenga, Aymeric Silvin, Garett Dunsmore, Yacine Haddad, Eugenie Pizzato, Pierre Ly, Caroline Flament, Cléa Melenotte, Eric Solary, Michaela Fontenay, Gabriel Garcia, Corinne Balleyguier, Nathalie Lassau, Markus Maeurer, Claudia Grajeda-Iglesias, Nitharsshini Nirmalathasan, Fanny Aprahamian, Sylvère Durand, Oliver Kepp, Gladys Ferrere, Cassandra Thelemaque, Imran Lahmar, Jean-Eudes Fahrner, Lydia Meziani, Abdelhakim Ahmed-Belkacem, Nadia Saïdani, Bernard La Scola, Didier Raoult, Stéphanie Gentile, Sébastien Cortaredona, Giuseppe Ippolito, Benjamin Lelouvier, Alain Roulet, Fabrice Andre, Fabrice Barlesi, Jean-Charles Soria, Caroline Pradon, Emmanuelle Gallois, Fanny Pommeret, Emeline Colomba, Florent Ginhoux, Suzanne Kazandjian, Arielle Elkrief, Bertrand Routy, Makoto Miyara, Guy Gorochov, Eric Deutsch, Laurence Albiges, Annabelle Stoclin, Bertrand Gachot, Anne Florin, Mansouria Merad, Florian Scotte, Souad Assaad, Guido Kroemer, Jean-Yves Blay, Aurélien Marabelle, Frank Griscelli, Laurence Zitvogel, Lisa Derosa

**Affiliations:** 1 Université Paris-Saclay, Faculté de Médecine, Le Kremlin-Bicêtre, France; 2 Gustave Roussy Cancer Campus, Villejuif, France; 3 Institut National de la Santé et de la Recherche Médicale, UMR1015, Gustave Roussy, Villejuif, France; 4 Département d’Oncologie Médicale, Gustave Roussy, Villejuif, France; 5 Département d’Innovation Thérapeutique et d’Essais Précoces, Gustave Roussy, Villejuif, France; 6 Département de Biostatistique et d’Epidémiologie, Gustave Roussy, Université Paris-Saclay, Villejuif, France; 7 Institut National de la Santé et de la Recherche Médicale Oncostat, U1018, Equipe labellisée par la Ligue Contre le Cancer, Gustave Roussy, Villejuif, France; 8 Médecine du travail, Gustave Roussy, Villejuif, France; 9 Institut Pasteur, Unit Biology and Genetics of the Bacterial Cell Wall, Paris, France; 10 CNRS UMR2001, Paris, France; 11 INSERM, Equipe Avenir, Paris, France; 12 Institut National de la Santé et de la Recherche Médicale, U1287, Gustave Roussy, Villejuif, France; 13 Département d’Hématologie, Gustave Roussy, Villejuif, France; 14 Université de Paris, Institut Cochin, Centre National de la Recherche Scientifique UMR8104, Institut National de la Santé et de la Recherche Médicale, Paris, France; 15 Service d’hématologie biologique, Hôpital Cochin, Assistance Publique—Hôpitaux de Paris.Centre-Université de Paris, Paris, France; 16 Département d’Imagerie Médicale, Gustave Roussy, Villejuif, France; 17 Biomaps, UMR1281, INSERM, CNRS, CEA, Université Paris Saclay, Paris, France; 18 Immunotherapy/Immunosurgery, Champalimaud foundation, Lisboa, Portugal; 19 Centre de Recherche des Cordeliers, Equipe labellisée par la Ligue contre le cancer, Université de Paris, Sorbonne Université, Inserm U1138, Institut Universitaire de France, Paris, France; 20 Metabolomics and Cell Biology Platforms, Gustave Roussy Cancer Center, Université Paris Saclay, Villejuif, France; 21 Institut National de la Santé et de la Recherche Médicale, U1030, Gustave Roussy, Villejuif, France; 22 Univ Paris Est Creteil, INSERM U955, IMRB, Creteil, France; 23 Service de maladies infectieuses, Centre Hospitalier de Cornouaille, Quimper, France; 24 Aix-Marseille Université, Institut de Recherche pour le Développement, Assistance Publique – Hôpitaux de Marseille, Microbes Evolution Phylogeny and Infections, Marseille, France; 25 Institut Hospitalo-Universitaire Méditerranée Infection, Marseille, France; 26 Aix Marseille Univ, School of medicine—La Timone Medical Campus, EA 3279: CEReSS—Health Service Research and Quality of life Center, Marseille, France; 27 Aix Marseille Université, IRD, AP-HM, SSA, VITROME, Marseille, France; 28 Scientific Direction, National Institute for Infectious Diseases Lazzaro Spallanzani, Rome, Italy; 29 Vaiomer, Labège, France; 30 Institut National de la Santé et de la Recherche Médicale, U981, Gustave Roussy, Villejuif, France; 31 Aix Marseille University, CNRS, INSERM, CRCM, Marseille, France; 32 Centre de ressources biologiques, ET-EXTRA, Gustave Roussy, Villejuif, France; 33 Département de Biologie Médicale et Pathologie Médicales, service de biochimie, Gustave Roussy, Villejuif, France; 34 Département de Biologie Médicale et Pathologie Médicales, service de microbiologie, Gustave Roussy, Villejuif, France; 35 Singapore Immunology Network, Agency for Science, Technology and Research (A*STAR), Singapore, Singapore; 36 Shanghai Institute of Immunology, Shangai, China; 37 Translational Immunology Institute, SingHealth Duke-NUS Academic Medical Center, Singapore, Singapore; 38 Cedar’s Cancer Center, McGill University Healthcare Centre, Montreal, QC Canada; 39 Centre de recherche du Centre hospitalier de l’Université de Montréal (CRCHUM), Montreal, QC Canada; 40 Department of Hematology-Oncology, Centre hospitalier de l’Université de Montréal, Montreal, QC Canada; 41 Institut National de la Santé et de la Recherche Médicale, U1135, Centre d’Immunologie et des Maladies Infectieuses, Hôpital Pitié-Salpêtrière, Assistance Publique—Hôpitaux de Paris, Paris, France; 42 Département de Radiothérapie, Gustave Roussy, Villejuif, France; 43 Service de Réanimation Médicale, Gustave Roussy, Villejuif, France; 44 Service de Pathologie Infectieuse, Gustave Roussy, Villejuif, France; 45 Service de médecine aigue d’urgence en cancérologie, Gustave Roussy, Villejuif, France; 46 Département Interdisciplinaire d’Organisation des Parcours Patients, Gustave Roussy, Villejuif, France; 47 Centre Léon Bérard, Lyon, France; 48 Université Claude Bernard, Lyon, France; 49 Unicancer, Paris, France; 50 Université de Paris, Paris, France; 51 Department of Women’s and Children’s Health, Karolinska Institute, Karolinska University Hospital, Stockholm, Sweden; 52 Pôle de Biologie, Hôpital Européen George Pompidou, Assistance Publique—Hôpitaux de Paris, Paris, France; 53 Suzhou Institute for Systems Biology, Chinese Academy of Medical Sciences, Suzhou, China; 54 Center of Clinical Investigations BIOTHERIS, Gustave Roussy, Villejuif, France; 55 Institut National de la Santé et de la Recherche Médicale—UMR935/UA9, Université Paris-Saclay, Villejuif, France; 56 INGESTEM National IPSC Infrastructure, Université de Paris-Saclay, Villejuif, France; 57 Université de Paris, Faculté des Sciences Pharmaceutiques et Biologiques, Paris, France

**Keywords:** Prognostic markers, Medical research

## Abstract

Patients with cancer are at higher risk of severe coronavirus infectious disease 2019 (COVID-19), but the mechanisms underlying virus–host interactions during cancer therapies remain elusive. When comparing nasopharyngeal swabs from cancer and noncancer patients for RT-qPCR cycle thresholds measuring acute respiratory syndrome coronavirus-2 (SARS-CoV-2) in 1063 patients (58% with cancer), we found that malignant disease favors the magnitude and duration of viral RNA shedding concomitant with prolonged serum elevations of type 1 IFN that anticorrelated with anti-RBD IgG antibodies. Cancer patients with a prolonged SARS-CoV-2 RNA detection exhibited the typical immunopathology of severe COVID-19 at the early phase of infection including circulation of immature neutrophils, depletion of nonconventional monocytes, and a general lymphopenia that, however, was accompanied by a rise in plasmablasts, activated follicular T-helper cells, and non-naive Granzyme B^+^FasL^+^, Eomes^high^TCF-1^high^, PD-1^+^CD8^+^ Tc1 cells. Virus-induced lymphopenia worsened cancer-associated lymphocyte loss, and low lymphocyte counts correlated with chronic SARS-CoV-2 RNA shedding, COVID-19 severity, and a higher risk of cancer-related death in the first and second surge of the pandemic. Lymphocyte loss correlated with significant changes in metabolites from the polyamine and biliary salt pathways as well as increased blood DNA from Enterobacteriaceae and Micrococcaceae gut family members in long-term viral carriers. We surmise that cancer therapies may exacerbate the paradoxical association between lymphopenia and COVID-19-related immunopathology, and that the prevention of COVID-19-induced lymphocyte loss may reduce cancer-associated death.

## Introduction

Severe acute respiratory syndrome coronavirus-2 (SARS-CoV-2) is a novel beta-coronavirus that has caused a worldwide pandemic of the human respiratory illness COVID-19, resulting in a severe threat to public health and safety worldwide. Because of age, gender, cancer-associated risk factors, metabolic syndrome, and side effects induced by their specific therapies (such as cardiomyopathy, systemic immunosuppression, and cellular senescence), cancer patients appear more vulnerable to severe infection than individuals without cancer [1]. Indeed, a high hospitalization and mortality rates of SARS-CoV-2 infection were heralded in patients with malignancy in several studies across distinct geographical sites [2–5]. Cancer types, performance status, and stage are additional risk factors for severe COVID-19 in this patient population. Patients with hematological, lung and breast cancer have been reported to be more susceptible to hospitalization or death due to COVID-19 as compared to patients with other malignancies [3, 5–8]. Patients diagnosed with metastatic cancers are more vulnerable to severe forms of COVID-19 than individuals with localized malignancies [9]. Recent (<3 months) cancer treatments including surgery, chemotherapy, and immunotherapy independently contribute to worsening the prognosis of COVID-19 among patients with the malignant disease [2, 5, 7, 9–11].

Here, we explored several independent cohorts of cancer patients diagnosed with COVID-19 (1063 patients, 58% with cancer) during the first surge of the pandemic to analyze the dynamics between host (blood immunology, metabolism, metagenomics) and viral parameters and validated the most clinically relevant findings in the second surge of the pandemic. We concluded that virus-induced or -associated lymphopenia that coincided with T-cell exhaustion, abnormalities in polyamine and biliary salt pathways and circulation of Enterobacteriaceae and Micrococcaceae bacterial DNA, is a dismal prognosis factor in cancer patients, likely participating in the vicious circle of immunosuppression-associated chronic virus shedding.

## Results

### Prolonged viral shedding and higher viral loads in cancer patients compared with cancer-free COVID-19^+^ patients

**Fig. 1:**
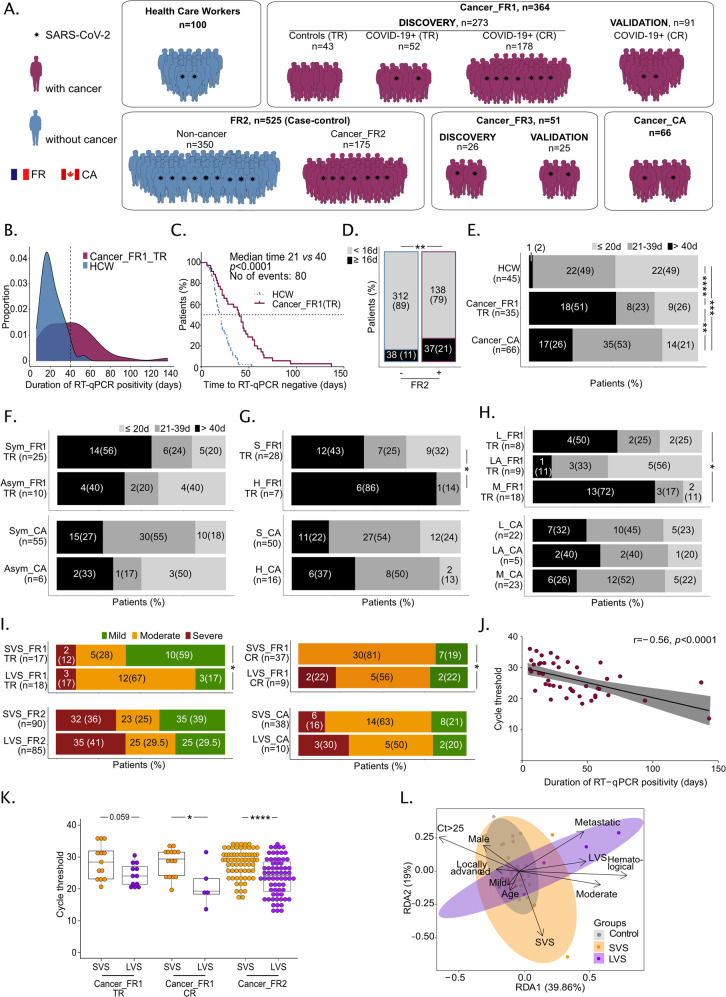
Prolonged duration of SARS-CoV-2 RNA shedding correlated with high viral load and COVID-19 severity in patients with cancer. **A** Graphical schema of cohorts and patients’ accrual. **B** Proportion of patients with cancer from translational research (TR) (Cancer_FR1_TR, *n* = 35, magenta area) or healthcare workers (HCW, *n* = 45, blue area) by days of RT-qPCR positivity. Vertical dashed line at 40 days represents the 95th percentile of HCW and the median of positivity of patients with cancer. **C** Kaplan–Meier curves of time to negative RT-qPCR in HCW (*n* = 45, blue dotted lines) and patients with cancer (Cancer_FR1_TR, *n* = 35, magenta continuous lines). **D** COVID-19^+^ cancer-bearing or history of cancer (+) and cancer-free (−) individuals from FR2 treated with hydroxychloroquine +/− azithromycin: number (percentages) of patients with RT-qPCR positivity beyond 16 days (90th percentile of the cancer-free population of FR2). **E** Number (percentages) of HCW, Cancer_FR1 patients (Cancer_FR1_TR), or Canadian patients with cancer (Cancer_CA) with short, intermediate (grouped in short-term viral RNA shedding, SVS), and prolonged (long-term viral RNA shedding, LVS) viral RNA shedding (**E**), according to the presence/absence of viral symptoms (symptomatic, Sym, vs asymptomatic, Asym) (**F**), diagnosis of hematological (H) versus solid (S) malignancy (**G**), and cancer staging (localized (L), locally advanced (LA), metastatic (M)) (**H**). **I** Number (percentages) of Cancer_FR1 patients (from translational research and clinical routine), Cancer_FR2 patients (Cancer_FR2) or Canadian patients with cancer (Cancer_CA) divided in SVS and LVS and regarding their respective COVID-19 severity. **J** Spearman correlation between Cycle threshold (Ct) for the RT-qPCR amplification of genes encoding proteins of SARS-CoV-2 replication–transcription complex at diagnosis and duration of RT-qPCR positivity for Cancer_FR1 (from translational research and clinical routine), each dot representing one sample/patient. **K** Ct values for the RT-qPCR amplification of genes encoding proteins of SARS-CoV-2 replication–transcription complex in nasopharyngeal swabs performed at diagnosis in SVS versus LVS in Cancer_FR1_TR and CR and Cancer_FR2, and dynamics over time from day 0 up to day 80 after inclusion in SVS (*n* = 33 samples, *n* = 28 patients, orange dots) versus LVS (57 samples, *n* = 17 patients, purple dots) in Cancer_FR1 (from translational research and clinical routine). **L** Redundancy statistical analysis (RDA) of cancer and viral related-clinical factors accounting for the variance of SARS-CoV-2 viral shedding status. Clinical components were influenced by the virus shedding (SVS versus COVID-19-negative, *P* = 0.037; LVS versus COVID-19 negative, *P* = 0.0010), COVID severity (mild versus COVID-19-negative, *P* = 0.0030; moderate versus COVID-19-negative, *P* = 0.0574; severe versus COVID-19-negative, *P* not computable), age (*P* = 0.0514), hematological rather than solid malignancy (hematological versus solid, *P* = 0.001), metastatic status (*P* = 0.0059), and Ct values at diagnosis (≥25 versus < 25, *P* = 0.0738). Chi-square tests with *P < 0.05, **P < 0.01, ***P < 0.001, ****P < 0.0001.

To explore the clinical significance of viral and/or immunological parameters in cancer patients, we gathered the data from electronic clinical files from various cancer centers or general hospitals across France and Canada, in order to monitor the magnitude and duration of virus RNA shedding in nasopharyngeal swabs according to cancer (versus healthcare workers (HCW) or COVID-19^+^ cancer-free individuals), tumor types (hematological versus solid malignancies) and staging (localized, locally advanced, metastatic diseases) (Fig. 1A). First, we conducted a prospective epidemiological study named Cancer_FR1_Translational Research (TR) at Gustave Roussy, Villejuif, France, during the first surge of the COVID-19 pandemic (from April 10, 2020 to May 11, 2020, NCT04341207) to evaluate the prevalence and severity of COVID-19 in all adult patients under treatment or recently treated for solid tumors or hematological malignancies (Fig. S1 and Table S1). Our secondary endpoint was the identification of viral, immunological, metabolic, and metagenomics blood predictors of severe complications among cancer patients. Clinical characteristics were collected from electronic medical records (Table S1). Nasopharyngeal samples were serially collected at every hospital visit motivated by the cancer management or any symptomatology related to seasonal flu or COVID-19 and transferred to the virology laboratory for SARS-CoV-2-specific quantitative reverse transcription-PCR (RT-qPCR) testing. Out of 473 patients enrolled in Cancer_FR1_TR, 53 (11%) were diagnosed with COVID-19 by RT-qPCR, and this diagnosis was corroborated by a specific serology in 87% of cases (Fig. S1A). Among the 52 patients evaluable for translational research, 37% were males, 60% suffered from at least one of the comorbidities associated with coronavirus pandemic, such as hypertension (58%) or obesity (21%) (Table S1). Seventy-seven percent had an ECOG performance status of 0–1 at the time of nasopharyngeal sampling. Twenty-one percent of COVID-19-positive cancer patients did not report any symptom of infection, 61% required hospital admission (for any cause or because of COVID-19 aggravation within 28 days after diagnosis) and 11% a transfer to intensive care unit (ICU), culminating with cancer death in 7% of the cases (from an undetermined cause, no systematic necropsy) (Fig. S1B–G and Table S1). Among patients with cancer diagnosed with COVID-19, 20% were followed up for hematological (as opposed to solid) malignancies and developed more severe symptoms of infection (Fig. S1B, G and Table S1). In the Cancer_FR1_TR study, 33%, 21%, and 46% presented with localized, locally advanced, and metastatic disease, respectively, that were equally susceptible to severe COVID-19 (Fig. S1F–G).

Given that cycle threshold (Ct) values of the first RT-qPCR test may be correlated with the clinical characteristics of the patients [12, 13], we performed a longitudinal follow-up of Ct values by RT-qPCR. We targeted several genes coding for the envelope, the nucleocapsid and/or the replication–transcription complex (RdRP, Orf1a, subgenomic RNA of the SARS-CoV-2 [14, 15]) to assess the duration of the nasopharyngeal SARS-CoV-2 RNA shedding, starting at COVID-19 diagnosis for up to 6 months as per-protocol indications (Fig. S2A). The duration of viral shedding was defined as the number of days from the first positive to the first negative RT-qPCR, after longitudinal monitoring with an interval inferior to 40 days, to reduce bias in viral shedding estimation. This time lapse of 40 days corresponded to the median of SARS-CoV-2 virus carriage in the cancer population (Fig. 1B, C and Table S1). In parallel, a similar and systematic COVID-19 protocol with longitudinal RT-qPCR testing was applied to healthcare workers (HCW) at Gustave Roussy. Healthcare workers had a mean age of 35 years (range: 19–61), were mostly females (male versus female: 13% versus 87%), and presented with one or two comorbidities in 27% and 4%, respectively, thereby significantly diverging from the cancer population diagnosed with COVID-19. Starting from 50 COVID-19-positive cancer patients and 100 HCW, we conducted RT-qPCR in 210 and 200 nasopharyngeal swabs, respectively (Fig. S2). However, applying the exclusion criteria detailed in Fig. S2, we could compare the median length of SARS-CoV-2 RNA detection in 35 cancer patients (Cancer_FR1_TR) and 45 HCW using 168 and 118 samples, respectively. Patients with cancer exhibited prolonged nasopharyngeal RNA virus shedding (Fig. 1B, median of 40 days (range: 6–137) for patients with cancer compared to 21 days (range: 7–53) for HCW, Fig. 1C, log-rank test *P* value < 0.0001). This difference persisted after adjusting for age, gender, and comorbidities (Cox multivariate analysis, adjusted hazard ratio (95% confidence interval) = 2.88 [1.42;5/85], *P* = 0.00291, Fig. 1C). To further validate the differences observed in the duration of viral RNA shedding between Cancer_FR1_TR and HCW, we analyzed another cohort of patients diagnosed with COVID-19 in a general hospital from Southern France and paired—in a case-control study—175 cancer patients (with a history of cancer or currently treated with cancer (Table S1)) with 350 cancer-free individuals based on age, gender, comorbidities, and COVID-19 severity (FR2_Case-Control, Cancer and Non-Cancer) (Fig. 1A and Table S1). Here again, there was a prolonged length of RT-qPCR positivity in cancer individuals compared with cancer-free COVID-19 patients (8 days versus 6 days, log-rank test *P* value, *P* = 0.03), taking into account that >70% were treated with hydroxychloroquine and azithromycin, a combination regimen reducing viral shedding [16]. Moreover, the proportion of patients with a viral shedding above 16 days (corresponding to the 90th percentile of the viral shedding in cancer-free patients) was higher in cancer patients (Fig. 1D, *P* < 0.0015). A second independent validation was achieved in the third series of 66 patients with cancer extracted from a cohort of 252 cancer individuals living in Canada and diagnosed with COVID-19 (Cancer_CA), for whom a longitudinal SARS-CoV-2-specific RT-qPCR (using *Orf1* and *E* gene probe sets [17]) follow-up had been carried out [18] (Fig. 1A and Table S1). Here again, we observed that 26% of cancer patients were still PCR positive after 40 days from diagnosis by RT-qPCR (Fig. 1E). Such a long-term PCR detection of viral RNA could indicate stable subgenomic RNA contained within double-membrane vesicles or the presence of a replicative mucosal viral strain. Hence, we confirmed in three independent series of cancer patients prolongation of RNA virus shedding previously described in case reports in hematological or immunocompromised patients [19–22].

Hence, we focused on the differential characteristics of cancer patients presenting with long-term viral RNA shedding (LVS), defined by a positive RT-qPCR duration ≥40 days (median of RT-qPCR duration in Cancer_FR1_TR (Fig. 1C)), compared to those experiencing Short term Viral RNA Shedding (SVS), defined by a positive RT-qPCR duration <40 days henceforth (Table S1). The increased susceptibility to develop a LVS was independent of initial symptomatology, observed in 33% of Canadian (CA) to 40% of French (FR1_TR) asymptomatic and 27% (CA) to 56% (FR1_TR) of symptomatic cancer patients (Fig. 1F). There was a higher propensity to LVS in hematological malignancies compared to solid cancers (86% versus 43%, respectively (*P* = 0.04, Fig. 1G and Table S1) and in advanced disease (*P* = 0.011) in FR1_TR cohort (Fig. 1H and Table S1) but less so, in the CA cohort. Importantly, the LVS phenotype was associated with an increased risk to develop a moderate form of COVID-19 (defined by thoracic CT scan, hospitalization, and oxygen requirement <9 L/min) in Cancer_FR1_TR (*P* = 0.032) (Fig. 1I). This trend was confirmed in the third series of French patients from the clinical routine (CR) managed outside the translational ancillary study at Gustave Roussy (called henceforth “Cancer_FR1_CR”; Table S1 and Fig. S3), where 20% of cancer patients were diagnosed with LVS and exhibited more severe COVID-19 infections (Fig. 1I, *P* = 0.011). Again, the hospitalization rates and transfer to intensive care units were increased in LVS compared with SVS patients in Cancer_FR1_TR (*P* = 0.0018, Table S1) and Cancer_FR2, respectively (*P* = 0.02, Table S1). Finally, the FR2 and Canadian series of LVS cancer patients also tended to exhibit more severe manifestations of COVID-19 compared with SVS Canadian cancer patients (Fig. 1I, bottom).

Of note, the duration of viral RNA shedding correlated with “viral load”, i.e., Ct values at diagnosis, in that cancer patient with LVS experienced lower Ct values at diagnosis than SVS cancer patients in most cohorts for which the data were available (Fig. 1J). Importantly, cancer patients doomed to develop LVS presented with lower Ct values at diagnosis than those prone to become SVS in Cancer_FR1 and Cancer_FR2 cohorts (Fig. 1K). Of note, Ct values at disease onset were significantly anticorrelated with duration of viral RNA shedding in cancer patients using either *N* or *Orf1ab*/*RdRP* gene-specific probe sets (data not shown).

The redundancy analysis (RDA) is an extension of the principal component analysis (PCA) aimed at identifying viral components which depend on other known covariates such as clinical parameters. RDA revealed that, within 30 days from diagnosis, 18% of the variance of the biological parameters are explained by ten components adjusted for the major clinical parameters for COVID-19 in Cancer_FR1_TR (Fig. 1L). These components were mainly influenced by the virus shedding (SVS versus COVID-19-negative, *P* = 0.037; LVS versus COVID-19-negative, *P* = 0.0010), COVID severity (mild versus COVID-19-negative, *P* = 0.0030; moderate versus COVID-19-negative, *P* = 0.0574; severe versus COVID-19-negative, *P* not computable), age (*P* = 0.0514), hematological rather than solid malignancy (hematological versus solid, *P* = 0.001), metastatic status (*P* = 0.0059), and Ct values at diagnosis (>25 versus < 25, *P* = 0.0738). As outlined in Table S1, LVS patients tended to be older (66 versus 56 years old, *P* = 0.08), more metastatic (72% versus 29%, *P* = 0.01), and experienced increased hospitalization rates (83% versus 23%, *P* < 0.001) than SVS cancer patients in the Cancer_FR1_TR cohort.

### Immunological hallmarks of long-term virus carriers at diagnosis

**Fig. 2:**
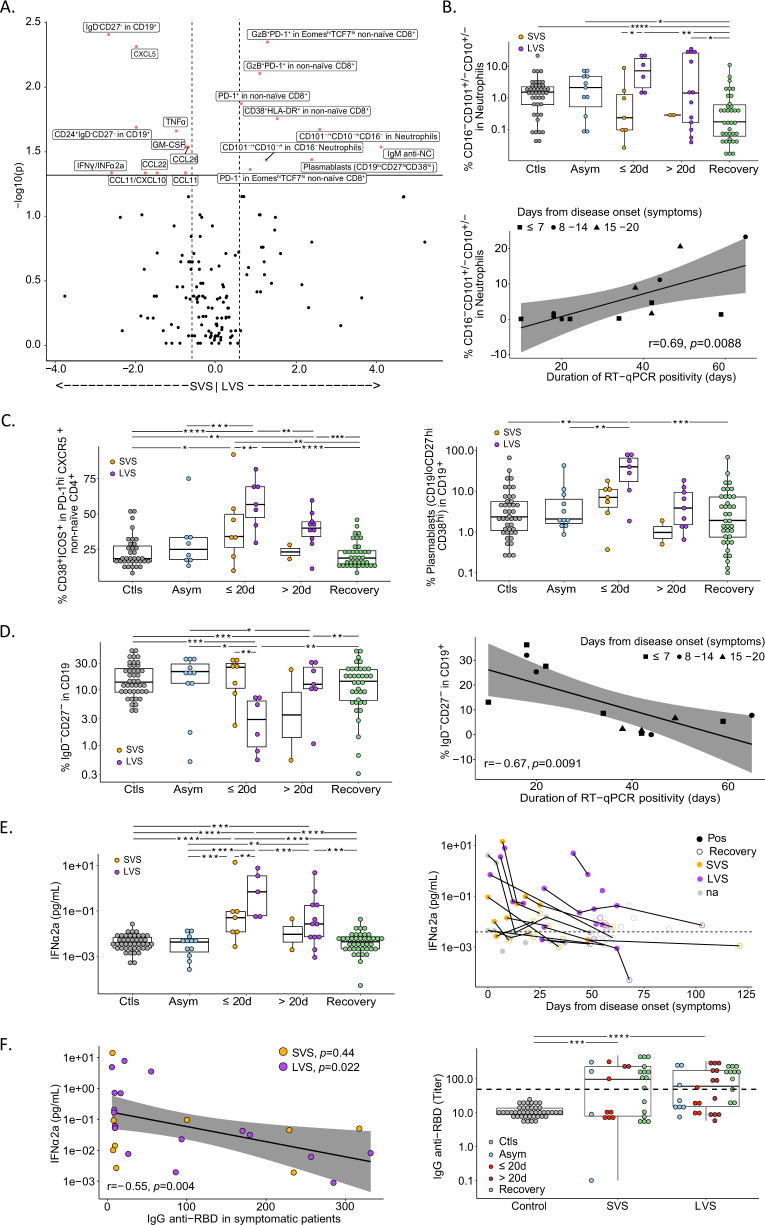
Immunotypes associated with prolonged viral RNA shedding in patients with cancer. **A** Volcano plot of the differential cellular and soluble immune parameters contrasting short-term viral RNA shedding (SVS) versus long-term viral RNA shedding (LVS) during the first 20 days of symptoms. Volcano plot was generated computing for each immune factor: (i) the log2 of fold change among the mean relative percentages after normalization in SVS versus LVS (*x* axis); (ii) the log10 of *P* values deriving from Wilcoxon test calculated on relative percentages in absolute values (*y* axis). Black and red dots are considered nonsignificant (*P* < 0.05) or significant (*P* > 0.05), respectively. **B**–**F** Temporal changes and correlation of blood leukocyte parameters measured by high-dimensional spectral flow cytometry (**B**–**D**) and soluble factors IFNα2a and anti-SARS-CoV-2 IgG (**E**, **F**) in various phases of COVID-19 presentation (no virus infection (Ctls, gray dots), asymptomatic viral infection (Asym, light blue dots), symptomatic viral infection examined in the first 20 days (≤20 d) or after 20 days (>20 d) of symptoms with those experiencing short-term viral RNA shedding (SVS, orange dots) or long-term viral RNA shedding (LVS, purple dots) and RT-qPCR-negative COVID-19 patients in the convalescent phase (recovery, green dots, or circled dots). Box plots display a group of numerical data through their 3rd and 1st quartiles (box), mean (central band), minimum and maximum (whiskers). Each dot represents one sample, each patient being drawn one to three times. Statistical analyses used one-way ANOVA with Kenward–Roger method to take into account the number of specimen/patient: *P < 0.05, **P < 0.01, ***P < 0.001, ****P < 0.0001. **B**–**D** Percentages of neutrophils that do not express either CD101 and/or CD10 and lost CD16 within the gate of CD45^+^CD56^-^CD3^-^CD19^-^CD15^+^ cells (**B**, upper panel). Spearman correlation between the percentage of immature neutrophils (CD10^+/−^CD101^+/−^CD16^−^) measured within the first 20 days of symptoms with the duration of SARS-CoV-2 RT-qPCR positivity (**B**, lower panel). **C**, **D** Percentages of CD38^+^ICOS^+^ among CXCR5^+^PD-1^+^ non-naive CD4^+^ (**C**, left panel), plasmablasts defined as CD19^low^CD38^high^CD27^+^ within the CD19^+^ gate (**C**, right panel), double-negative IgD^-^CD27^-^ among CD19^+^ cells (**D**, left panel) and their Spearman correlation when measured within the first 20 days of symptoms with the duration of SARS-CoV-2 RT-qPCR positivity (**D**, right panel). **E** Ultrasensitive electrochemiluminescence assay to monitor the serum concentrations of IFNα2a (**E**, left panel) in a kinetic fashion (**E**, right panel). Each line and dot represent one patient and one sample, respectively, and the dashed line represents the median value of controls. **F** Spearman correlation between the serum IFNα2a values measured in symptomatic patients with IgG titers against SARS-CoV-2 S1 RBD considered as continuous variables (**F**, left panel). The raw data are represented in the right panel at both time points for each group of patients.

Intrigued by these findings, we addressed the question as to whether and how prolonged viral RNA shedding would impact on Cancer_FR1_TR patients with respect to COVID-19-related immunological alterations previously reported for cancer-free infected individuals [23–29]. More than 80 phenotypic markers were quantified on circulating leukocytes by means of high-dimensional spectral flow cytometry, complemented by multiplex ELISAs to detect serum chemokines, cytokines, and growth factors. These parameters were recorded within or after the first 20 days of inclusion in the Cancer_FR1_TR protocol, for 25 COVID-19^+^ cancer patients that were divided into LVS versus SVS subgroups, in comparison to 43 COVID-19-negative cancer patients (“controls” or “Ctls”) matched for age, gender, comorbidities, cancer types, and tumor extension (Table S2). Asymptomatic individuals and cancer patients enrolled at the recovery phase of COVID-19 (meaning that they became PCR-negative) were analyzed separately. Within the first 20 days from diagnosis, LVS presented increased proportions of monocytes among circulating leukocytes (Fig. S4A, left panel), and a parallel drop in CD169^-^HLA-DR^+^ within conventional monocytes (Fig. S4A, middle panel) and in nonconventional monocytes (CD16^+^CD14^low/-^, Fig. S4A, right panel) compared to SVS, cancer controls, asymptomatic or recovered patients, as reported [23, 30]. Polymorphonuclear cells (PMN) tended to increase in LVS, specifically immature CD101^+/−^CD10^+/−^CD16^−^ neutrophils, compared with SVS, convalescent, and controls (Fig. 2A, B, upper and lower panels and Fig. S4B).

Importantly, the most significant phenotypic traits distinguishing LVS from SVS featured among the reported hallmarks of severe COVID-19 in cancer-free subjects [23–29] (Fig. 2A). In accordance with the reported defects in germinal center formation in secondary lymphoid organs of severe COVID-19 [28], LVS cancer patients exhibited increased recirculation of activated CXCR5^+^PD-1^high^ CD4^+^ follicular T-helper cells (TFH) expressing ICOS and CD38 (Fig. 2C, left panel), as well as a marked rise in plasmablasts (defined as CD19^low^ CD27^hi^ CD38^hi^) at the expense of transitional B (CD24^+^CD38^hi^CD19^+^) and double-negative B cells (IgD^-^CD27^-^CD19^+^) (Fig. 2C, right panel, Fig. S4C and Fig. 2D). As indicated in the Volcano plot in Fig. 2A, immature PMN and double-negative B cells were among the most significant immunological features, positively and negatively predicting LVS, respectively (Fig. 2B, bottom panel and Fig. 2D, right panel). LVS coincided with the prolonged systemic release of, and exposure to, type 1 IFN above levels measured in SVS, controls, and recovered individuals (Fig. 2E). Type 1 IFN levels anticorrelated with titers of neutralizing anti-S1 RBD antibodies (Fig. 2F). This landscape of immune profiling was corroborated by non-supervised hierarchical clustering of innate and cognate immunotypes and serum cytokine concentrations analyzed within 30 days from diagnosis. This method allowed to segregate a small cluster of individuals characterized by low Ct values (<25), and moderate/severe complications of COVID-19, which included metastatic cancer carriers with LVS or SVS (Fig. S5). This cluster was separated from the others by typical signs of viral infection, including abundant circulating CD38^+^HLA-DR^+^CD8^+^T cells, plasmablasts, activated TFH cells, and high serum IFNα2a levels (Fig. S5). Likewise, while many inflammatory cytokines, chemokines, or alarmins (such as IFNγ, CXCL10, IL-4, IL-6, and calprotectin) were elevated in symptomatic COVID-19 individuals compared with controls, asymptomatic, and recovered patients, none of them could predict LVS, except a drop in the IFNγ/IFNα2a and CCL11/CXCL10 ratios whose significance remains unclear (*P* = 0.016 and *P* = 0.0019, respectively) (Fig. S4D–I). Interestingly, innate and cognate immunotypes performed in convalescent patients and controls segregated at random in the non-supervised hierarchical clustering (Fig. S6).

Altogether, the high-dimensional flow cytometry of blood immune subsets indicated that LVS cancer patients harbored the immunological hallmarks of severe COVID-19 at diagnosis.

### Virus-associated lymphopenia predicted shorter overall survival in the first and second surge of the pandemic

**Fig. 3:**
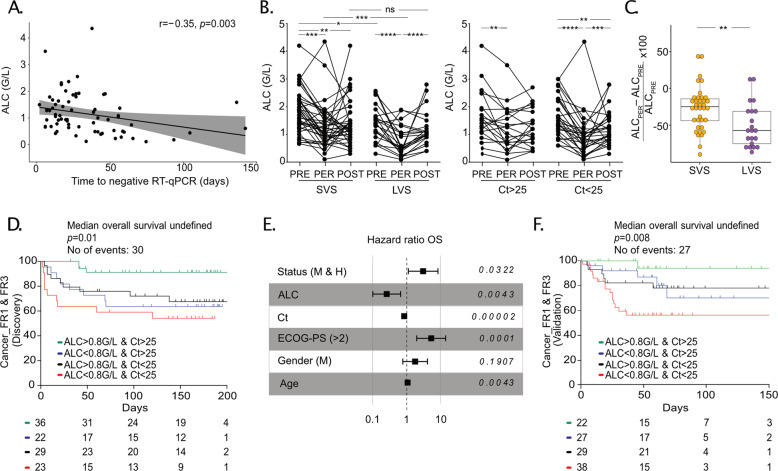
Lymphopenia and high viral load are dismal prognosis factors for overall survival in cancer patients in the first and second surge of the pandemic. **A** Spearman correlation between the absolute lymphocyte counts (ALC) of Cancer_FR1 (from translational research and clinical routine), with the duration of SARS-CoV-2 RT-qPCR positivity (only evaluable patients for both factors, *n* = 69 patients). **B**, **C** ALC of Cancer_FR1 (from translational research and clinical routine) in SVS (*n* = 37 patients) versus LVS (*n* = 22 patients) subsets (**B**, left panel) or SARS-CoV-2-cycle threshold (Ct) >25 (*n* = 21 patients) versus Ct <25 (*n* = 29 patients) (**B**, right panel) monitored during the COVID-19 pandemic (“PER”, between −4 and +7 days of the disease diagnosis by RT-qPCR), between 210 and 12 days before the symptom onset of COVID-19 (“PRE”) or within the recovery period (between 0 and 123 days after negative RT-qPCR) (“POST”) at Gustave Roussy, with the calculation of the reduction between “PRE” and during COVID-19 (**C**). One patient defined as an outlier (at 215%) by ROUT method was excluded from the LVS group for the analysis. Each line and dot represents one patient and one sample. Statistical analyses used one-way ANOVA (paired and unpaired) with Kenward–Roger method taking into account the number of specimen/patient (**B**): *P < 0.05, **P < 0.01, ***P < 0.001, ****P < 0.0001, and Mann–Whitney (**C**): **P < 0.01. **D** Kaplan–Meier curve and Cox regression analysis of overall survival of cancer patients from the Discovery (1st surge) cohort (Cancer_FR1 + Cancer_FR3), all stages included, according to ALC and Ct value at diagnosis. Refer to Table 1 for patient characteristics. **E** Multivariate Cox regression analysis stratified for the cohort and adjusted for age, ECOG status, gender, and metastatic and/or hematological status of cancer patients from the Discovery (1st surge) cohort (Cancer_FR1 + Cancer_FR3). **F** Kaplan–Meier curve and Cox regression analysis of overall survival of cancer patients from Validation (2nd surge) cohort (Cancer_FR1 + Cancer_FR3), all stages included, according to ALC and Ct value at diagnosis. Refer to Table 1 for patient characteristics.

Lymphocyte loss is a feature of severe COVID-19 in noncancer patients [24, 27]. The “FR2” cohort was a case-control study with 175 cancer patients paired with 350 cancer-free individuals based on age, gender, comorbidities, and COVID-19 severity. As observed in Fig. 1J for cancer patients, there was an anti-correlation between Ct values at diagnosis and the duration of viral RNA shedding in cancer-free patients (*r* = −0.6, *P* < 0.0001) (Fig. S7A). Not surprisingly, blood absolute lymphocyte counts (ALC) at diagnosis anticorrelated with the duration of PCR positivity in Cancer_FR1_TR and Cancer_FR1_CR cohorts (Fig. 3A). However, although the ALC before the COVID-19 pandemic (blood drawn from December 2019 to mid-March 2020) were already somewhat lower in LVS than in SVS cancer patients, the ALC during the outbreak dramatically dropped in cancer patients doomed to develop LVS (in both Cancer_FR1_TR and Cancer_FR1_CR cohorts), more so than in individuals prone to SVS (Fig. 3B, left panel). The extent in ALC reduction was more severe in patients presenting LVS than SVS (Fig. 3C). Of note, ALC recovered in both patient groups regardless of the LVS/SVS status. It supports that reduced ALC at COVID-19 diagnosis is induced by the virus rather than by cancer (Fig. 3B, left panel). In accord with the finding that LVS correlates with high viral load at symptom onset (Fig. 1J, K), higher viral loads at diagnosis were associated with a pronounced COVID-19-associated lymphopenia (Fig. 3B, right panel). Contrary to what we observed in cancer patients, there was no correlation between ALC at diagnosis, and duration of RT-qPCR in cancer-free individuals (*r* = 0.05, *P* = 0.3) (Fig. S7B). Comparing ALC at diagnosis to ALC post-hospitalization, we concluded that cancer-free patients presenting with a high viral load (Ct<25) did not harbor lymphopenia at diagnosis or during the acute phase (*P* = 0.11) (Fig. S7C) in contrast with what we observed in cancer patients. So, virus-induced lymphocyte loss occurs in a fraction of individuals with cancer and is detrimental for the prognosis. This phenomenon may be ascribed to cancer-associated chronic inflammation or co-medications.

We next assessed the clinical significance of the interaction between Ct values, ALC, and cancer patient survival in 110 cancer patients with COVID-19 (Discovery cohort (first surge of the pandemic) including 84 patients from Cancer_FR1 treated at Gustave Roussy and 26 patients from Cancer_ FR3 treated at Léon Bérard Cancer Center in Lyon, France) (Fig. 3D and Table 1). Cox logistic regression analyses and Kaplan–Meier survival curves were performed after stratification of the patients according to both, Ct and ALC values at diagnosis. The cutoff for the Ct value was 25 and corresponded to the median of the whole cohort FR1 + FR3, which coincided with the threshold at which live virus particles can be isolated in 70% of the cases [31]. The cut-off value for ALC was the median found in patients with high viral load (Ct <25) at diagnosis (ALC = 800/mm^3^). ALC combined with Ct values predicted cancer-related overall survival in univariate analyses across all cancer stages (local, locally advanced, or metastatic) (Fig. 3D and Table 1). While patients presenting with ALC > 800/mm^3^ and low viral load (Ct >25) exhibited prolonged survival, a dismal prognosis affected 21% of them (23/110) who presented both deep lymphopenia (ALC < 800/mm^3^) and high viral loads (Ct <25) at diagnosis (Fig. 3D) culminating in 40% deaths at 3 months. All four groups were comparable in terms of age, gender, comorbidities, cancer type, or staging (Table 1). Multivariate Cox analysis stratified for the cohort origin and adjusted for age (hazard ratio (95% confidence interval) = 1.042 [1.013; 1.072], *P* = 0.0043), ECOG performance status (4.547 [1.845; 11.206], *P* = 0.0001), gender (1.668 [0.775; 3.588], *P* = 0.1907), and metastatic status and hematological malignancies (2.747 [1.090; 6.923], *P* = 0.0322) confirmed a continuous decrease of risk with the increase of the Ct value (0.841 [0.776; 0.911], *P* = 0.00002) and the increase of the ALC (0.282 [0.119; 0.672], *P* = 0.0043) (Fig. 3E). Of note, treatment retardation could not explain the high mortality of patients presenting with a high viral load and low ALC (Table 1).

**Table 1:** Clinical characteristics of Cancer_FR1 and Cancer_FR3 patients from discovery and validation cohorts presenting cycle threshold below (Ct<25) or above 25 (Ct>25) and with (<800/mm^3^) or without (>800/mm^3^) lymphopenia at diagnosis (refer Fig. 3D–F).

Discovery cohort							
Cancer patients’ characteristics		Cancer_FR1_TR + Cancer_FR1_CR + Cancer_FR3 (*n* = 110)	Ct > 25 & ALC > 800 (*n* = 36)	Ct > 25 & ALC < 800 (*n* = 22)	Ct < 25 & ALC > 800 (*n* = 29)	Ct < 25 & ALC < 800 (*n* = 23)	*P*
Age (year)	Median (range)	62 (13–95)	62 (13–82)	63 (20–83)	59 (38–95)	60 (21–84)	*0.76*^#^
Gender—no. (%)	Male	46 (42)	18 (50)	9 (41)	13 (45)	6 (26)	*0.33*
	Female	64 (58)	18 (50)	13 (59)	16 (55)	17 (74)	
Number of comorbidities —no. (%)°	0	38 (45)	10 (45)	5 (34)	10 (38)	13 (62)	*0.26*
	1	25 (30)	5 (23)	6 (40)	10 (38)	4 (19)	
	2	16 (19)	4 (18)	2 (13)	6 (24)	4 (19)	
	3	5 (6)	3 (14)	2 (13)	0 (0)	0 (0)	
Comorbidities —no. (%)°	COPD	6 (7)	2 (9)	1 (7)	1 (4)	2 (10)	*0.98*
	BMI ≥ 30	12 (14)	2 (9)	3 (20)	4 (15)	3 (14)	
	Hypertension	32 (38)	11 (50)	7 (47)	8 (31)	6 (29)	
	Congestive heart failure	3 (6)	1 (5)	1 (7)	1 (4)	0 (0)	
	Diabetes mellitus	10 (12)	3 (14)	1 (7)	4 (15)	2 (10)	
Type of malignancy —no. (%)	S	92 (84)	33 (92)	15 (68)	26 (90)	18 (78)	*0.08*
	H	18 (16)	3 (8)	7 (32)	3 (10)	5 (22)	
Cancer spread —no. (%)	Localized	19 (17)	7 (19)	1 (5)	7 (24)	4 (17)	*0.46*
	Locally advanced	24 (22)	9 (25)	6 (27)	3 (10)	6 (26)	
	Metastatic	67 (61)	20 (56)	15 (68)	19 (66)	13 (57)	
Cancer status —no. (%)	Remission or NED	29 (26)	12 (30)	3 (14)	10 (34)	4 (17)	*0.21*
	SD/PD	47 (43)	17 (47)	11 (50)	11 (38)	8 (35)	
	Present or PD	34 (31)	7 (19)	8 (36)	8 (28)	11 (48)	
ECOG PS—no. (%)	0	28 (25)	13 (36)	5 (23)	5 (18)	5 (22)	*0.01*
	1	46 (42)	18 (50)	4 (18)	12 (41)	12 (52)	
	2 or more	36 (33)	5 (14)	13 (59)	12 (41)	6 (26)	
Type of anticancer therapy—no. (%)	None*	53 (48)	20 (56)	8 (36)	14 (48)	10 (43)	*0.53*
	Chemotherapy	47 (43)	4 (11)	12 (55)	11 (38)	14 (61)	*0.19*
	Radiotherapy	8 (7)	2 (6)	3 (14)	1 (3)	2 (9)	
	Surgery	8 (7)	3 (8)	2 (9)	3 (10)	0 (0)	
	Hormonal therapy	11 (10)	4 (11)	0	4 (14)	3 (13)	
	Immunotherapy	12 (11)	4 (11)	1 (5)	4 (14)	3 (13)	
	Others	11 (10)	2 (6)	2 (9)	0 (0)	5 (22)	
Delay of treatment—no. (%)°	Yes (range: 16–170 days)	12 (32)	2 (33)	2 (22)	8 (67)	0 (0)	*<0.01*
	No	26 (68)	4 (67)	7 (78)	4 (33)	11 (100)	
Clinical course —no. (%)°	Day hospital	27 (32)	10 (45)	4 (27)	8 (31)	5 (24)	*0.63*
	Hospitalization	53 (63)	12 (55)	10 (67)	17 (65)	14 (67)	
	Admission to ICU	4 (5)	0	1 (6)	1 (4)	2 (9)	
Death—no. (%)	Yes	31 (28)	4 (11)	7 (32)	9 (31)	11 (48)	*0.02*

Validation cohort							
Cancer patients’ characteristics		Cancer_FR1_CR + Cancer_FR3 (*n* = 116)	Ct > 25 & ALC > 800 (*n* = 22)	Ct > 25 & ALC < 800 (*n* = 27)	Ct < 25 & ALC > 800 (*n* = 29)	Ct < 25 & ALC < 800 (*n* = 38)	*P*
Age (year)	Median (range)	65 (13–91)	55 (13–86)	64 (46–77)	68 (41–84)	66 (18–91)	*0.09*^#^
Gender—no. (%)	Male	71 (61)	9 (41)	14 (52)	17 (59)	23 (61)	*0.48*
	Female	45 (39)	13 (59)	13 (48)	12 (41)	15 (39)	
Type of malignancy —no. (%)	S	85 (73)	19 (86)	19 (70)	22 (76)	25 (66)	*0.36*
	H	31 (27)	3 (14)	8 (30)	7 (24)	13 (34)	
Cancer spread —no. (%)	Localized	9 (8)	3 (14)	1 (4)	3 (10)	2 (5.3)	*0.40*
	Locally advanced	15 (13)	4 (18)	6 (22)	3 (10)	2 (5.3)	
	Metastatic	82 (70)	15 (68)	17 (63)	21 (72)	29 (76.4)	
	Unknown	10 (9)	0 (0)	3 (11)	2 (8)	5 (13)	
Type of anticancer therapy—no. (%)°*	None*	33 (36)	14 (74)	8 (36)	5 (23)	6 (21)	*0.001*
	Chemotherapy	28 (31)	1 (5)	11 (50)	5 (23)	11 (39)	*0.17*
	Radiotherapy	5 (5)	0 (0)	2 (9)	1 (5)	2 (7)	
	Surgery	1 (1)	0 (0)	0 (0)	1 (5)	0 (0)	
	Hormonal therapy	4 (4)	2 (11)	1 (5)	0 (0)	1 (4)	
	Immunotherapy	20 (22)	2 (11)	2 (9)	6 (27)	10 (36)	
	Others	17 (19)	1 (5)	4 (18)	5 (23)	7 (25)	
Death—no. (%)	Yes	27 (23)	1 (4)	5 (18)	6 (21)	15 (39)	*0.016*

*P* values are in Italic and were analyzed by Chi-Square / Fisher’s exact tests.

*BMI* body mass index, *COPD* Chronic obstructive pulmonary disease, *CR* clinical routine, *Ct* cycle threshold, *DM* diabetes mellitus, *H* hematological malignancies, *ICU* intensive care unit, *n* number, *NED* no evidence of disease, *no.* number, *PD* progressive disease, *PS* performance status, *S* solid tumors, *SD/PR* stable disease/partial response, *TR* translational research, *in the 4 weeks before inclusion.

Statistical analyses: ANOVA (Kruskal–Wallis)(^#^), Chi-Square or Fisher’s exact tests.

°Unknown for Cancer_FR3_discovery (*n* = 26 patients), calculations with Cancer_FR1_discovery, *n* = 84.

°*Unknown for Cancer_FR3_validation (*n* = 25 patients), calculations with Cancer_FR1_validation, *n* = 91.

We confirmed these predictors (ALC < 800 & Ct <25) of poor survival during the second surge of the pandemic (between May 5, 2020 to November 25, 2020) in 116 new COVID-19 cancer patients (“Validation”, Cancer_FR1 and Cancer_FR3, Fig. 1A). Here again, the subset of patients with ALC < 800 & Ct <25 (*n* = 38/116, 32.7%) exhibited the most reduced overall survival compared to the other groups with >40% deaths at 50 days (Fig. 3F). Of note, the reduced survival rate in the subset of patients defined by ALC < 800 & Ct <25 was not a peculiarity of hematological malignancies (characterized by therapy-induced B cell depletion) since it was also observed in patients with solid neoplasia (Fig. S7D and S7E).

In conclusion, it appears that uncontrolled viral infection capable of compromising the number and function of circulating lymphocytes predicts the lethal outcome of patients with malignant disease.

### Immunological, metabolic, and metagenomic parameters associated with virus-induced lymphocyte loss

Multiple and non-exclusive mechanisms could account for virus-associated lymphopenia [25, 27, 32–35]. To further investigate this deleterious virus-induced lymphocyte loss, we searched for the most robust correlates between ALC and immunological, metabolic, or pathogenic cues in the Cancer_FR_TR cohort as well as noncancer COVID-19 patients that we previously reported [23].

**Fig. 4:**
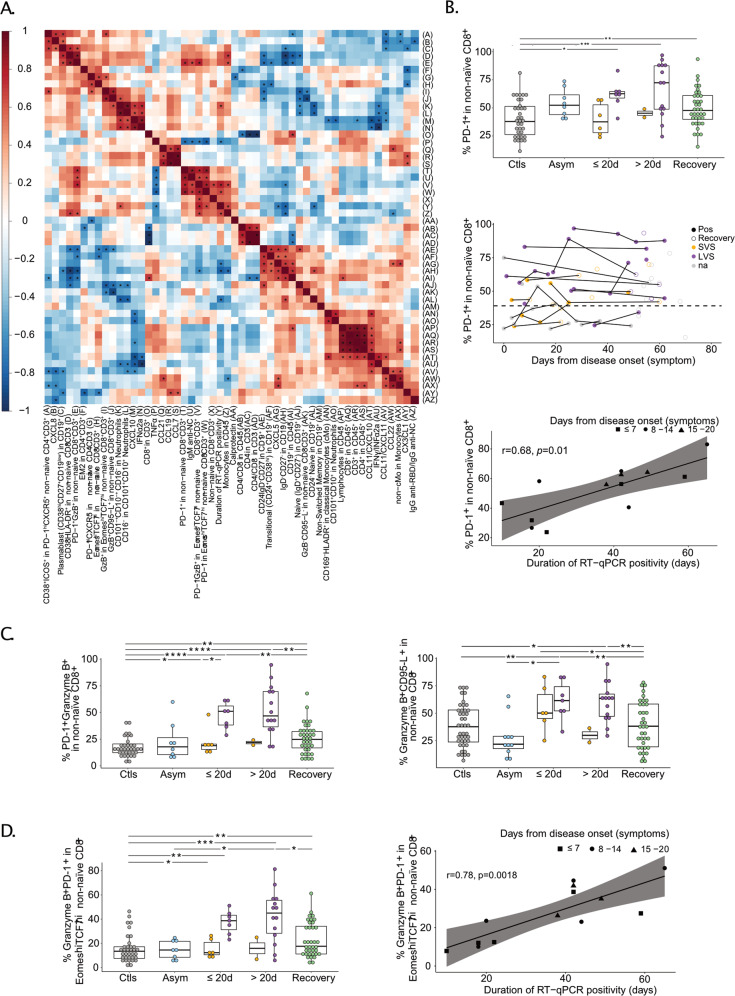
Prolonged viral shedding is associated with T-cell exhaustion. **A** Spearman correlation matrix focusing on the most significant immune variables and serum analytes monitored within the first 20 days of symptoms in patients diagnosed with COVID-19 in the Cancer_FR1_TR cohort. Stars indicate significant values (*P* < 0.05) for positive (red) or negative (blue) correlations. **B** Percentages of PD-1 expressing cells within the non-naive CD8^+^CD3^+^ population (**B**, upper panel), monitoring in various phases of COVID-19 presentation (no virus infection (Ctls, gray dots), asymptomatic viral infection (Asym, light blue dots), symptomatic viral infection examined in the first 20 days (≤20 d) or after 20 days (>20 d) of symptoms with those experiencing short-term viral RNA shedding (SVS, orange dots) or long-term viral RNA shedding (LVS, purple dots) and RT-qPCR-negative COVID-19 patients in the convalescent phase (recovery, green dots or circled dots) among Cancer_FR1_TR (**B**, middle panel) and Spearman correlation with the duration of SARS-CoV-2 RT-qPCR positivity measured within the first 20 days of symptoms (**B**, lower panel). **C** Percentages of subsets co-expressing PD-1 and Granzyme B (**C**, left panel) or Granzyme B and FasL (**C**, right panel) in non-naive CD8^+^. **D** Percentage of PD-1^+^ and Granzyme B^+^ within the non-naive CD8^+^ expressing Eomes^high^TCF-1^high^ gate (**D**, left panel) and Spearman correlation between this ratio measured within the first 20 days of symptoms with the duration of SARS-CoV-2 RT-qPCR positivity (**D**, right panel). Box plots display a group of numerical data through their 3rd and 1st quartiles (box), mean (central band), minimum, and maximum (whiskers). Each dot represents one sample, each patient being drawn one to three times. Statistical analyses used one-way ANOVA with Kenward–Roger method to take into account the number of specimen/patients: *P < 0.05, **P < 0.01, ***P < 0.001. Each line and dot represents one patient and one sample, respectively (**B**, middle panel).

First, the Spearman correlation matrix of the main immunological and serum markers monitored at the peak of disease (within the first 20 days of disease onset) indicated close interconnections between lymphocyte proportions and their subsets within leukocytes (Fig. 4A). Lymphopenia, which is a prominent feature of COVID-19 and a hallmark of severe infection, distinguished LVS from SVS or asymptomatic individuals (Fig. S8A, B), as exemplified for the proportion of B lymphocytes among total CD45^+^ leukocytes after 20 days of symptoms. As reported [27], the transitional differentiation of naive into effector/memory T cells co-expressing CD38^+^HLA-DR^+^ among CD8^+^T cells is a hallmark of COVID-19 that persisted in LVS compared to controls and SVS *(P* = 0.002 and *P* = 0.012*)* (Fig. S8C, D). In particular, the most compelling LVS-associated T-cell subpopulation that expanded in the context of lymphopenia was the non-naive (non-CD45RA^+^CD27^+^) CD8^+^ T subset expressing an activation/exhaustion phenotype characterized by early and sustained expression of PD-1 (Fig. 4B), Eomes, Granzyme B, TCF-1 including the pro-apoptotic marker CD95-L (FasL) (Fig. 4C, D, left panel). There was no difference in T-bet^+^ (effector) expression within Eomes^+^PD-1^+^ non-naive CD8^+^ over the different time courses and compared with controls (6.2 ± 0.74% (mean ± SEM) (data shown). However, COVID-19^+^ patients (both asymptomatic and symptomatic ones) exhibited higher proportions of cells co-expressing TOX and Eomes within PD-1^+^ non-naive CD8^+^ compared with patients at recovery or controls (Fig. S8E, left panel). Interestingly, a subset of these exhausted PD-1^+^CD8^+^ T cells was proliferating while undergoing apoptosis during the acute phase compared with patients at recovery (Fig. S8E, right panel). All of these data tend to indicate that circulating PD-1-expressing CD8^+^ T cells are rather exhausted than activated with a trend toward apoptosis that could participate in the lymphopenia described in COVID-19^+^ cancer patients. The abundance of these non-naive exhausted PD-1^+^CD8^+^ Tc1 cells positively correlated with the duration of SARS-CoV-2-specific RT-qPCR positivity (Fig. 4B, bottom panel and Fig. 4D, right panel) and may explain, at least partly, the reduced fitness and half-life of peripheral lymphocytes.

**Fig. 5:**
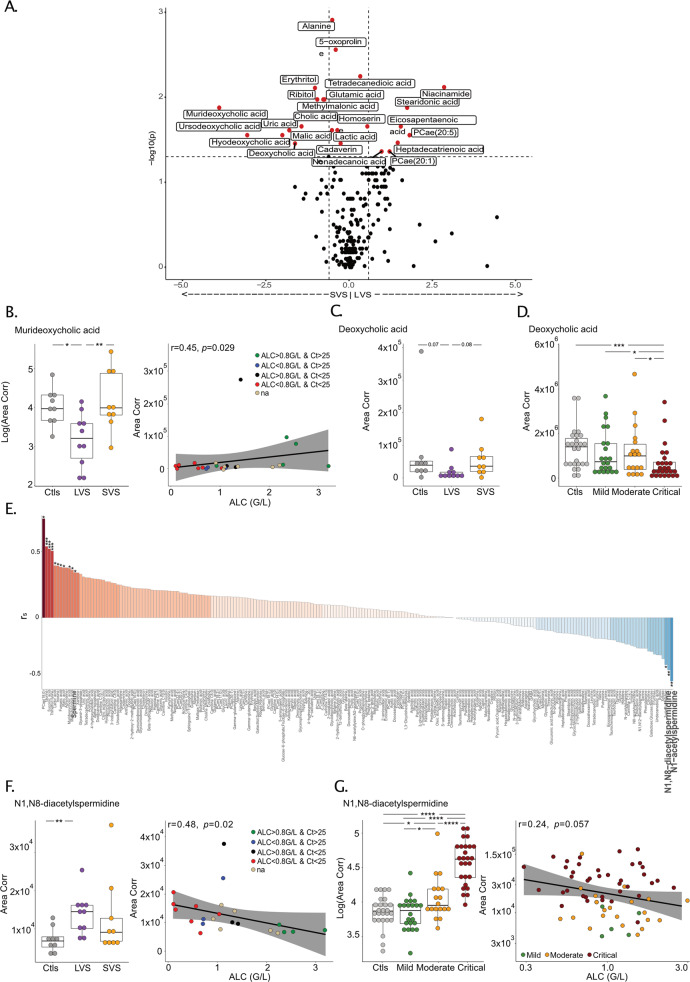
Lymphopenia and prolonged viral shedding are associated with perturbations of the polyamine and biliary acid pathways. **A** Volcano plot identifying statistically different serum metabolites between patients experiencing short-term viral RNA shedding (SVS) and those experiencing long-term viral RNA shedding (LVS) in Cancer_FR1_TR cohort. Metabolites significantly different between both groups are in red and annotated (*P* < 0.05, FC > 0.5). **B** Levels of murideoxycholic acid according to the duration of viral shedding in Cancer_FR1_TR (left panel) and Spearman correlation with absolute lymphocyte count (ALC) (right panel). The color code corresponds to the category of cycle threshold (Ct) and ALC at diagnosis. **C**, **D** Serum concentrations of deoxycholic acid according to the duration of viral shedding in Cancer_FR1_TR (**C**) and the severity of COVID-19 infection in cancer-free individuals (**D**). **E** Waterfall plot of Spearman’s correlation coefficient (r_s_) between ALC and 221 metabolites in the serum of patients diagnosed positive for COVID-19. **F** N1, N8 diacetylspermidine relative abundance in controls, SVS and LVS patients in the Cancer_FR1 cohort, that is negatively correlated with the ALC. The color code corresponds to the category of cycle threshold (Ct) and ALC at diagnosis. **G** Levels of N1, N8 diacetylspermidine in noncancer COVID-19 patients according to the clinical severity compared to COVID-19-negative controls (Ctls) (*P* < 0.0001) (**G**, left panel), that are negatively correlated with the absolute lymphocyte count (ALC) (**G**, right panel). Box plots display a group of numerical data through their 3rd and 1st quartiles (box), mean (central band), minimum and maximum (whiskers). Each dot represents one sample, each patient being drawn once for cancer-free individuals and one to two times for cancer patients. Statistical analyses used one-way ANOVA with Kenward–Roger method to take into account the number of specimen/patient (**B**, left panel, **C**–**E**, left panel): *P < 0.05, **P < 0.01), non-parametric unpaired Wilcoxon test (Mann–Whitney) for each two-group comparison: *P < 0.05, **P < 0.01, ***P < 0.001, ****P < 0.0001.

Second, we performed the serum metabolome determined by untargeted and targeted mass spectrometry-based metabolomics analyzing more than 221 metabolites in 31 cancer patients from Cancer_FR1_TR, as well as in a previously described cohort of 66 cancer-free COVID-19^+^ patients for validation [23]. The non-supervised hierarchical clustering of the serum metabolome clearly contrasted LVS from LVS patients (Fig. S9). The Volcano plot aimed at identifying significant differences between LVS and SVS patients pointed out the biliary salt metabolic pathway segregating SVS from LVS serum (Fig. 5A), previously described to have biological significance for lymphocyte fitness and maintenance [32–35]. Secondary biliary acids (such as the murideoxycholic acid (muri-DOC) (Fig. 5B, left panel) and the DOC (Fig. 5C)) were decreased in LVS compared with SVS and controls and correlated with lower ALC in cancer patients (Fig. 5B, right panels) or severe COVID-19 (Fig. 5D). Similarly, two other derivatives of DOC (hyo-DOC, urso-DOC) were decreased in LVS (compared to controls and SVS, Fig. S10A, B, left panels) and were associated with lymphocyte loss (Fig. S10A and S10B, right panels).

Another metabolic pathway pertaining to polyamines with high biological significance for age-related immunosenescence [36–38] was also strongly associated with the duration of RT-qPCR positivity, ALC, and disease severity (Fig. 5E–G and Fig. S9). In particular, the N1, N8 diacetylspermidine that anticorrelated with ALC (Fig. 5F, right panel) increased in the serum of LVS patients (but not SVS, Fig. 5F, left panel), in accordance with its marked rise in severe COVID-19 in cancer-free individuals (Fig. 5G, left panel) where high levels coincided with the lymphocyte drop (Fig. 5G, right panel). Of note, the tryptophane/kynurenine or lactic acid metabolites were not relevant in our study (Fig. 5A and Fig. S9).

**Fig. 6:**
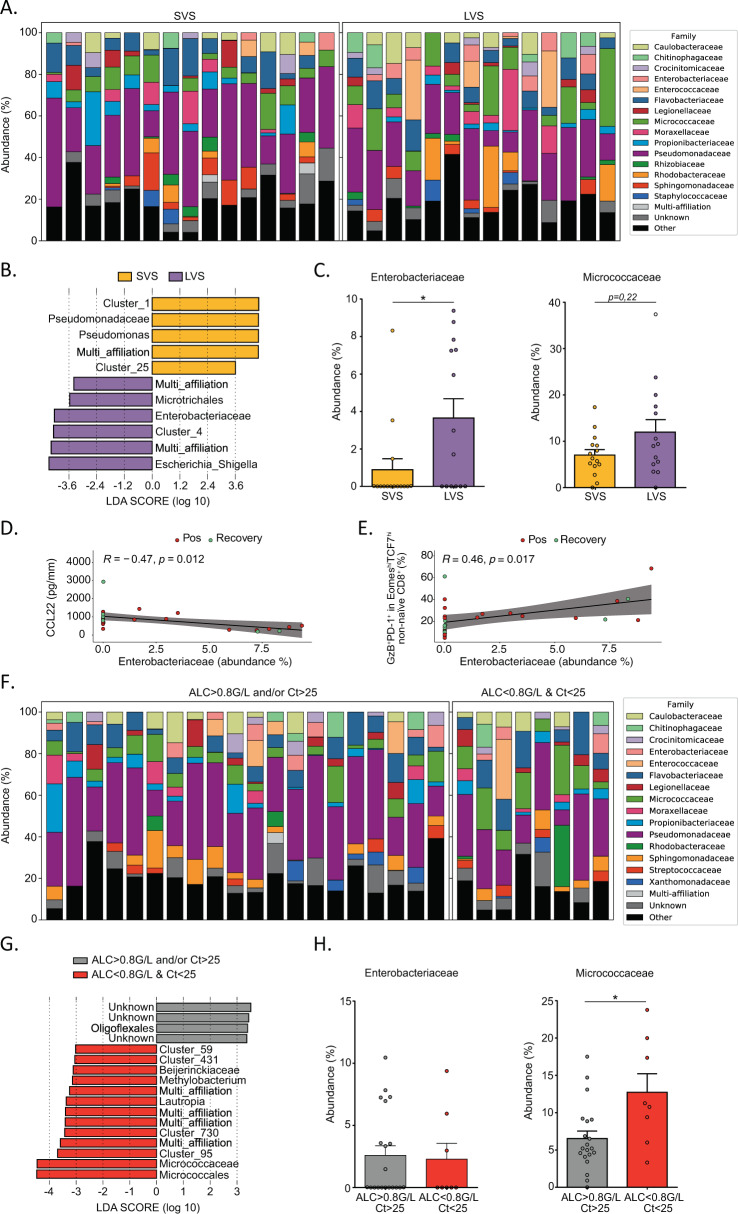
Lymphopenia and prolonged viral shedding are associated with blood recirculation of Enterobacteriaceae and Micrococcaceae DNA. **A** Stacked bar charts showing the relative abundance of bacterial families obtained by 16S sequencing of the whole-blood samples in patients experiencing short-term viral RNA shedding (SVS) and long-term viral RNA shedding (LVS) among Cancer_FR1_TR. Only the top 15 most abundant bacterial families are represented (the others are in the category “Other”). **B** Linear discriminant analysis effect size (LEfSe) analysis displaying linear discriminant analysis score (LDA) of the blood bacterial taxa differentially recovered from SVS (orange) versus LVS (purple) patients (*P < 0.05 with Mann–Whitney test between the two groups of patients). **C** Mean (bar plots, +/− SEM) and individual values (dot plots) of relative proportions of Enterobacteriaceae (**C**, left panel) and Micrococcaceae (**C**, right panel) family members in SARS-CoV-2-positive and recovered patients. Significance between SVS and LVS patients was evaluated using Mann–Whitney test (*P < 0.05). **D**, **E** Spearman correlations between the relative proportions of Enterobacteriaceae with paired concentrations of CCL22 in serum (**D**) and with paired percentages of Granzyme B (GzB)^+^PD-1^+^ in Eomes^hi^TCF-1^hi^ non-naive CD8^+^ measured in blood (**E**). **F** Idem as in **A**. considering segregating the cohort in two groups; ALC > 0.8 G/L and/or Ct >25 patients versus ALC < 0.8 G/L & Ct <25 patients. **G** LEfSe analysis displaying LDA score of the blood bacterial taxa significantly increased in ALC > 0.8 G/L and/or Ct >25 patients (gray) and ALC < 0.8 G/L & Ct <25 patients (red). The displayed bacterial taxa are significantly different (*P < 0.05 with Mann–Whitney test) between the two groups of patients. **H** Idem as in C segregating the cohort into the same two groups as in **F**. Significance between ALC > 0.8 G/L and/or Ct >25 patients and ALC < 0.8 G/L & Ct <25 patients was evaluated using the Mann–Whitney test (*P < 0.05).

Third, endotoxemia was shown to correlate with the cytokine storm during COVID-19 [25] and might cause activation-induced lymphocyte cell death. Assuming that the gut permeability could be altered during COVID-19-associated intestinal dysbiosis [39], we studied the circulating microbial populations associated with whole leukocytes by sequencing blood rDNA using next-generation sequencing of V3–V4 variable regions of the 16S rRNA bacterial gene as previously described [40]. Although we failed to observe significant quantitative differences in blood bacterial load between SVS (*n* = 14) and LVS (*n* = 15) patients, the linear discriminant analysis effect size indicated significant taxonomic differences in the bacteria family members between the two groups (Fig. 6A, B). The DNA from Enterobacteriaceae (mainly composed of *Escherichia Shigella* genus) was overrepresented in leukocytes of LVS compared with SVS patients (Fig. 6A, B, C, left panel). The circulating Enterobacteriaceae-related DNA markedly anticorrelated with CCL22 (a hallmark of SVS, Fig. 2A), but was strongly associated with the increase of exhausted CD8^+^ T lymphocytes (Fig. 6D, E). There was a trend for an increase in the relative abundance of Micrococcaceae in the blood leukocytes of LVS that was confirmed in cancer patients with dismal prognosis (ALC < 800 & Ct <25) (Fig. 6F, G, H).

Overall, we conclude that virus-associated lymphopenia may result in complementary or coordinated orthogonal disorders.

## Discussion

To interrogate viral–host interactions during the COVID-19 pandemic in cancer patients, we studied 1106 patients, among them 59% were cancer bearers (FR1 + FR2 + FR3 + CA), and 1063 COVID-19-positive (Fig. 1A). We used high-dimensional flow cytometry to perform deep immune profiling of innate, B and T cells, and measurements of 51 soluble markers, with temporal analysis of immune changes during infection in one cohort that was further explored by blood metabolomics and metagenomics. This longitudinal immune analysis was linked to virologic and oncological data (Figs. S5 and S6). Using this approach, we made several intriguing observations.

First, 51%, 20%, and 26% of cancer patients in FR1_TR, FR1_CR, and CA, respectively, still shed SARS-CoV-2 RNA after day 40 from symptoms onset (versus 2% in HCW), correlating with high viral loads (Ct values <25) at diagnosis. Indeed, isolation of replication-competent viral strains between 10 and 20 days after symptom onset has been documented in some persons with severe COVID-19, mostly in immunocompromised cases [41]. However, ~90% of their specimens no longer yielded replication-competent viruses after day 15 from symptom onset [42, 43]. Prolonged shedding of influenza, parainfluenza, rhinovirus, seasonal coronavirus, and the respiratory syncytial virus has previously been detected in immunosuppressed patients [44–48]. Cancer dissemination, cancer therapies, and virus-induced lymphopenia might cause an immunodeficiency that eventually jeopardizes virus clearance. The proposed mechanisms by which lymphopenia occurs in COVID-19 (often shared with cancer dissemination) [49] include virus-induced atrophy of secondary lymphoid organs [50–52], the disappearance of germinal centers [28], the direct pro-apoptotic activity of the virus related to ACE2-dependent or ACE2-independent entry into lymphocytes [53], T-cell demise consecutive to activation and exhaustion [54, 55], senescence [1, 56], and antiproliferative effects of lactic acid [57]. However, in our study, we found that lymphocyte loss was correlated with a decrease of secondary biliary salts in LVS patients, most likely associated with increased gut permeability that leads to bacterial translocation, as we observed increased circulating DNA for Micrococcaceae and Enterobacteriaceae family members. Moreover, the transformation of spermidine into N1, N8 diacetylspermidine was linked to decreased ALC, in accordance with the role of spermidine in preventing aging-related loss of lymphocyte fitness [36–38].

Second, prolonged viral RNA carriage was associated with signs of immunopathology (exacerbated T-cell responses, extrafollicular TFH, and plasmablast recirculation, exhausted PD-1^+^ Tc1 cells, sustained serum type 1 IFN levels), likely maintaining a positive feedback loop for the expression of the interferon-signaling genes product ACE2 [58] and pro-inflammatory interactions between airway epithelia and immune cells [29].

Third, prolonged SARS-CoV-2 RNA shedding after day 40 might precede the aggravation of both COVID-19 and malignant disease. Indeed, virus and/or cancer-induced lymphopenia and T-cell exhaustion may jointly enfeeble tumor immunosurveillance [59]. Interestingly, SARS-CoV-2 virus-induced immunopathology was accompanied by increased blood levels of IL-8 (Fig. S4G) and VEGF [26], which are well-known pro-angiogenic and pro-tumorigenic growth factors, predicting failure to cancer immunotherapy [60]. Of note, patients with high initial viral loads or LVS tended to accumulate poor prognosis-related parameters than SVS or patients with higher Ct values in both cohorts (Table 1 & Table S1), being older (66 versus 56 years old, *P* = 0.08), more metastatic at diagnosis of infection (72% versus 29%, *P* = 0.011), and increased hospitalization rates (83% versus 23%, *P* = 0.001). As a result, virus-induced lymphopenia markedly predicted early death of patients, within the first 2–3 months post-COVID-19 diagnosis in the first and second surge of the pandemic (in more than 200 patients) and call for caution to administer chemotherapy or steroids at the acute phase of the viral infection that exacerbate immunosuppression.

These observations call for a careful follow-up of cancer patients, in particular those bearing hematological and metastatic malignancies, during the second wave of COVID-19. Given the non-consensual efficacy of vaccines against influenza virus in vulnerable individuals suffering from cancer-, virus-, and age-associated lymphopenia [49, 61], passive immunization of high affinity neutralizing monoclonal antibodies against SARS-CoV-2 at COVID-19 onset might be envisaged. This could be combined with therapeutic stimulation of lymphopoiesis (for instance with rIL-7, G-CSF, inhibitors of indoleamine 2,3 deoxygenase), to achieve immunological tonus that is compatible with anticancer treatments [62–64]. Clinical trials are underway to evaluate rIL-7 against COVID-19, but may benefit from patient stratification based on Ct values, duration of viral RNA shedding, and ALC [65, 65, 66].

## Materials and methods

All cohorts (refer to Supplementary Material, Table 1).

### Cohorts for the duration of viral PCR positivity

#### Cancer_FR1_Translational Research (TR) (ONCOVID) clinical trial and regulatory approvals for translational research

*Principles:* Gustave Roussy Cancer Center sponsored the “ONCOVID” trial and collaborated with the academic authors on the design of the trial and on the collection, analysis, and interpretation of the data. Sanofi provided trial drugs. The trial was conducted in accordance with Good Clinical Practice guidelines and the provisions of the Declaration of Helsinki. All patients provided written informed consent. Protocol approval was obtained from an independent ethics committee (ethics protocol number EudraCT No: 2020-001250-21). The protocol is available with the full text of this article at https://clinicaltrials.gov/ct2/show/NCT04341207. *Patients:* ONCOVID eligible patients were adults fitted for, or under, or recently treated by chemotherapy and/or immune-checkpoint blockade for the treatment of solid tumors or hematological malignancies (please refer to Table 1 and Table S1). Patients diagnosed for COVID-19 from April 10, 2020 to May 4, 2020 were included in the Discovery cohort and patients from May 5, 2020 to November 25, 2020 were included in the Validation cohort. *Trial design:* Cancer patients were screened for SARS-CoV-2 virus carriage by nasopharyngeal sampling at every hospital visit. The presence of SARS-CoV-2 RNA was detected by RT-qPCR assay in a BSL-2 laboratory. Asymptomatic and symptomatic patients (i.e.*,* presenting with fever (*t*°>38 °C) and/or cough and/or shortness of breath and/or headache and/or fatigue and/or runny nose and/or sore throat, anosmy/agueusia) with a positive SARS-CoV-2 RT-qPCR test, shifted to the interventional phase (tailored experimental approach with hydroxychloroquine and azithromycin therapy in symptomatic SARS-CoV-2-positive subjects). Asymptomatic or symptomatic patients with negative SARS-CoV-2 RT-qPCR test continued their standard of care anticancer treatments. Repeated RT-qPCR for SARS-CoV-2 on nasopharyngeal swabs and blood samples were performed to monitor the status for SARS-CoV-2 and the immune response, respectively, in COVID-19-positive and negative patients. The COVID-19 severity was defined based on oxygen, imaging, and hospitalization criteria. Patients with mild COVID-19 disease had limited clinical symptoms not requiring scan or hospitalization; patients with a moderate COVID-19 disease were symptomatic with dyspnea and radiological findings of pneumonia on thoracic scan requiring hospitalization and a maximum of 9 L/min of oxygen; severe patients had respiratory distress requiring intensive care and/or more than 9 L/min of oxygen. *Samples for translational research:* Whole blood was used for high-dimensional spectral flow cytometry analyses. Serum samples were used to monitor the concentrations of cytokines and chemokines released and to titer anti-SARS-CoV-2 IgG, M and A antibodies (see “Blood analysis” section) (Supplementary Material Fig. 1).

#### Healthcare workers (HCW) of Cancer_FR1

The part of the research including healthcare workers was conducted in compliance with General Data Protection Regulation (GDPR) and the French Data Protection Authority’s recommendation about Data Protection in clinical researches. Gustave Roussy Data Protection Officer (DPO) has evaluated this project and sent to the principal investigator a formalized operational action plan about data protection compliance: patient’s information, security measures, good practices about pseudonymization, etc. All of the DPO’s recommendations have been applied by the research team. Healthcare workers diagnosed for COVID-19 between 24 March, 2020 and 24 April, 2020 were included. Results of RT-qPCR, cycle threshold, age, gender, and number of comorbidities were collected. Data from healthcare workers who refused to participate and/or with cancer were excluded. In agreement with MR004 in France, we reported the series to the national information science and liberties commission.

#### Second series of patients with cancer (Cancer_FR2)

*CASE-CONTROL study:* All comers spontaneously presenting at a general hospital for infectious diseases (IHU Méditerranée Infections, Marseille, FR) (Table S1) from February 27, 2020 to December 15, 2020 composed of 996 COVID-19 patients. We performed a case-control study at a 1:2 paired ratio where the 175 cancer patients (with a currently treated cancer or history of cancer) were matched with 350 cancer-free individuals on age, gender, comorbidities relevant for COVID-19. Of note, >75% received hydroxychloroquine and >96% received azithromycin (Table S1) [16, 67]. This study was approved by the IHU Méditerranée Infections review board committee (Méditerranée Infection N°: 2020-021).

#### Third series of cancer patients from Canada (Cancer_CA)

We used 66 individuals from the clinical cohort previously reported [18] for whom data were available (Table S1). This study was conducted across eight Canadian institutions in Quebec and British Columbia and was approved by the institutional ethics committee at each site (Ethics number: MP-02- 2020-8911 and H20-00892).

#### Fifth series of cancer patients, Cancer_FR1_Clinical Routine (CR)

We used the clinical cohort previously reported [2] (Table S1). In accordance with the French regulations, there was no requirement for ethical approval to be sought for this observational study, based on medical files. Patients diagnosed for COVID-19 from March 14, 2020 to April 29, 2020 were included in the Discovery cohort and from April 29, 20 to November 25, 2020 in the Validation cohort. This study was also declared to the Gustave Roussy Cancer Centre’s DPO and registered on the website of the French Healthcare Data Institute (declaration number: MR4911200520).

### Cohorts for the ALC and Ct value predictors: first surge and the second surge of the pandemic

#### Cancer_FR1_Translational Research (TR) (ONCOVID) clinical trial and regulatory approvals for translational research

Among the 52 patients diagnosed for COVID-19 during the first surge (from April 10, 2020 to May 4, 2020), absolute lymphocyte count (ALC) and cycle threshold (Ct) were available for 34 patients whom were included in this cohort.

Then, among the 18 patients included in ONCOVID during the second surge (from May 5, 2020 to November 25, 2020), absolute lymphocyte count (ALC) and cycle threshold (Ct) were available for nine patients who were included in this cohort

#### Cancer patients referred to the clinical routine (Cancer_FR1_CR)

In accordance with the French regulations, there was no requirement for ethical approval to be sought for this observational study, based on medical files. Among the 178 patients diagnosed for COVID-19 during the first surge (March 14, 2020 to April 29, 2020), ALC and Ct were available for 50 patients who were included in this cohort. Then, among 170 patients with cancer diagnosed for COVID-19 during the second surge (from May 5, 2020 to November 25, 2020), ALC and cycle threshold Ct were available for 82 patients who were included in this cohort.

#### Cancer patients referred to the Centre Léon Bérard, Lyon, France (Cancer_FR3)

The PRE-ONCOVID-19 study was approved by the Institutional review board of the Centre Leon Bérard on March 12, 2020 (ET20-069). We used a subset of 25 patients included during the first surge from March 5, 2020 to May 4, 2020 with available ALC and Ct values. We used 26 patients included during the second surge from October 1, 2020 to December 5, 2020 with available data.

Patients from each cohort were classified using the same criteria.

### RT-qPCR analysis

SARS-CoV-2 diagnostic testing of clinical nasopharyngeal swabs or other samples by RT-qPCR was conducted from March 14, 2020 to March 23, 2020 at an outside facility using the Charité protocol. From March 23, 2020 testing was performed internally at the Gustave Roussy. The cycle thresholds were collected only for assays performed at Gustave Roussy. Nasopharyngeal swab samples were collected using flocked swabs (Sigma Virocult) and placed in viral transport media. SARS-CoV-2 RNA was detected using one of two available technics at Gustave Roussy: the GeneFinder COVID-19 Plus Real*Amp* kit (ELITech Group) targeting three regions (*RdRp* gene, nucleocapsid, and envelope genes) on the ELITe InGenius (ELITech Group) or the multiplex real-time RT-PCR diagnostic kit (the Applied Biosystems TaqPath COVID-19 CE-IVD RT-PCR Kit) targeting three regions (*ORF1ab*, nucleocapsid and spike genes) with the following modifications. Nucleic acids were extracted from specimens using automated Maxwell instruments following the manufacturer’s instructions (Maxwell RSC simply RNA Blood Kit; AS1380; Promega). Real-time RT-PCR was performed on the QuantiStudio 5 Dx Real-Time PCR System (Thermo Fisher Scientific) in a final reaction volume of 20 μl, including 5 μl of extracted nucleic acids according to the manufacturer instruction.

The cut-off value of 25 for the cycle threshold was based on the median calculated on Cancer_FR1_TR and the mean calculated on Cancer_FR1_TR + CR.

### RT-PCR for subgenomic RNA (sgRNA) for SARS-CoV-2

We used the protocol previously described by Wölfel et al. [15]. Briefly, the oligonucleotide sequence of the leader-specific primer was as follows: sgLeadSARSCoV2-F; CGATCTCTTGTAGATCTGTTCTC, and the oligonucleotide sequence of the E primer was as follows: E_Sarbeco_R; ATATTGCAGCAGTACGCACACA. Briefly, 5 uL of RNA (>21 ng) were used for the sgRNA RT-PCR assay with Superscript III one-step RT-PCR system with Platinum Taq Polymerase (Invitrogen, Darmstadt, Germany) with 400 nM concentration of each primer. Thermal cycling was set up as described. Finally, RT-PCR products for sgRNAs were analyzed on agarose gel 2%.

### Evaluation of SARS-CoV-2 RNA shedding

The duration of viral shedding was defined as the number of days from the first positive to the first negative RT-qPCR, after longitudinal monitoring. In order to prevent an overvaluation of this duration, we considered in this analysis only patients with an interval below 40 days between the last positive RT-qPCR and the first negative RT-qPCR. Six patients had one negative RT-qPCR followed by positive RT-qPCR. We extend the duration to the second negative RT-qPCR for three patients with a cycle threshold below 35 for the gene coding replication–transcription complex and within 6 days after the first negative result.

### Absolute lymphocyte count (ALC)

The absolute lymphocyte count was measured for the clinical routine using the Sysmex XN (Sysmex, Belgium). Values “PRE” were collected between 210 and 12 days before the symptom onset of COVID-19, values at diagnosis of the infection were collected between −4 and +7 days of the disease diagnosis by RT-qPCR, values “POST” were collected at the recovery time or later, meaning between 0 and 123 days after the first negative RT-qPCR. For the interpretation, the cut-off value for ALC was the median found in patients with high viral load at diagnosis (ALC = 800/mm^3^). In parallel, we considered this value as relevant according to the common terminology criteria for adverse events where grades of lymphopenia were assigned as follows: grade 1 ALC < lower limit of normal to 800/mm^3^, grade 2 ALC < 800–500/mm^3^, and grade 3 ALC < 500–200/mm^3^.

### Blood tests

#### Sampling

Blood samples were drawn from patients enrolled in ONCOVID at Gustave Roussy Cancer Campus (Villejuif, France). Whole human peripheral blood was collected into sterile vacutainer tubes.

#### Spectral flow cytometry

One hundred and twenty-one whole-blood samples from 88 patients (Supplementary Material Fig. 1) were mixed at a 1:1 ratio with Whole Blood Cell Stabilizer (Cytodelics), incubated at room temperature for 10 min and transferred to −80 °C freezer to await analysis. These samples were secondarily thawed in a water bath set to +37 °C. Cells were fixed at a ratio 1:1 with Fixation Buffer (Cytodelics, ratio 1:1) and incubated for 10 min at room temperature. Red blood cells were lysed by the addition of 2 mL of Lysis Buffer (Cytodelics, ratio 1:4) at room temperature for 10 min. White blood cells were washed with 2 mL of Wash Buffer (Cytodelics, ratio 1:5). Cells were resuspended in 100 µL extracellular antibody cocktail and incubated at room temperature for 15 min, then washed in Flow Cytometry Buffer (PBS containing 2% of fetal bovine serum and 2 mM EDTA). For intracellular labeling, a step of permeabilization was performed using 200 µL of eBioscience Foxp3 kit (ThermoFischer); cells were then incubated for 40 min at +4 °C, washed in Perm Buffer (ThermoFischer) and resuspended in an intracellular antibody cocktail. After incubation, cells were washed in Flow Cytometry Buffer and resuspended to proceed to the acquisition. All antibodies used are listed in Supplemental Material Table 2. Samples were acquired on CyTEK Aurora flow cytometer (Cytek Biosciences)(Cytek Biosciences) (T cell, B cell and myeloid cell/global panels) or BD LSR Fortessa X20 Flow cytometer (BD Biosciences-US)(apoptosis and exhaustion panel).

### Data analysis

16S rDNA metagenomic profiling DNA from 100 µL of whole blood (from 5 mL EDTA sampling tube) was isolated and amplified in a strictly controlled environment at Vaiomer SAS (Labège, France) using a stringent contamination-aware approach, as discussed previously [40, 68–70]. The microbial populations based on rDNA present in whole blood were determined using next-generation sequencing of V3–V4 variable regions of the 16S rRNA bacterial gene as previously described [69]. For each sample, a sequencing library was generated by the addition of sequencing adapters. The joint pair length was set to encompass a 467 base pairs amplicon (using Escherichia coli 16S as a reference) with a 2 × 300 paired-end MiSeq kit V3 (Illumina, San Diego, CA, USA). The detection of the sequencing fragments was performed using the MiSeq Illumina® technology. Targeted metagenomic sequences from microbiota were analyzed using the bioinformatic pipeline from the FROGS guideline [71]. Briefly, the cleaning was done by removing amplicons without the two PCR primers (10% of mismatches were authorized), amplicons with at least one ambiguous nucleotide (“N”), amplicons identified as chimera (with vsearch v1.9.5), and amplicons with a strong similarity (coverage and identity ≥80%) with the phiX (library used as a control for Illumina sequencing runs). Clustering was produced in two passes of the swarm algorithm v2.1.6. The first pass was a clustering with an aggregation distance equal to 1. The second pass was a clustering with an aggregation distance equal to 3. Taxonomic assignment of amplicons into operational taxonomic units (OTUs) was produced by Blast+ v2.2.30+ with the Silva 134 Parc databank. To assess if the richness of microbiota was adequately captured by metagenomic sequencing, a rarefaction analysis was performed. To ensure a low background signal from bacterial contamination of reagents and consumables, two types of negative controls consisting of molecular grade water were added in an empty tube separately at the DNA extraction step and at the PCR steps and amplified and sequenced at the same time as the extracted DNA of the blood samples. The controls confirm that bacterial contamination was well contained in our pipeline and had a negligible impact on the taxonomic profiles of the samples of this study as published before [40, 68–70]. One sample has been excluded from the analyses for the aberrant profile.

### Serum tests

Serums from 120 samples corresponding to 88 patients (Supplementary Material Fig. 1) were collected from whole blood after centrifugation at 600 × *g* for 10 min at room temperature and transferred to −80 °C freezer to await analysis.

#### Multiplex cytokine and chemokine measurements

Serum samples were centrifuged for 15 min at 1000 × *g*, diluted 1:4, then monitored using the Bio-Plex ProTM Human Chemokine Panel 40-plex Assay (Bio-rad, ref: 171AK99MR2) according to the manufacturer’s instructions. 40-plex cytokines and chemokines provided are CCL1, CCL11, CCL13, CCL15, CCL17, CCL19, CCL2, CCL20, CCL21, CCL22, CCL23, CCL24, CCL25, CCL26, CCL27, CCL3, CCL7, CCL8, CX3CL1, CXCL1, CXCL10, CXCL11, CXCL12, CXCL13, CXCL16, CXCL2, CXCL5, CXCL6, CXCL8, CXCL9, GM-CSF, IFN-γ, IL-10, IL-16, IL-1β, IL-2, IL-4, IL-6, MIF, TNF- α. Acquisitions and analyses were performed on a Bio-Plex 200 system (Bio-rad) and a Bio-Plex Manager 6.1 Software (Bio-rad), respectively. Soluble Calprotectin (diluted 1:100) and IFN-α2a were analyzed using a R-plex Human Calprotectin Antibody Set (Meso Scale Discovery, ref: F21YB-3) and the ultrasensitive assay S-plex Human IFN-α2a kit (Meso Scale Discovery, ref: K151P3S-1), respectively, following manufacturer’s instructions. Acquisitions and analyses of soluble Calprotectin and IFN-α2a were performed on a MESO™ QuickPlex SQ120 reader and the MSD’s Discovery Workbench 4.0. Each serum sample was assayed twice with the average value taken as the final result.

#### Serology: anti-SARS-CoV-2 immunoglobulins

Serum was collected from whole blood after centrifugation at 600 × *g* for 10 min at room temperature and transferred to −80 °C freezer to await analysis. Serological analysis SARS-CoV-2-specific IgA, IgM, and IgG antibodies were measured in 119 serum samples from 87 patients (Supplementary Material Fig. 1) with The Maverick ™ SARS-CoV-2 Multi-Antigen Serology Panel (Genalyte Inc. USA) according to the manufacturer’s instructions. The Maverick™ SARS-CoV-2 Multi-Antigen Serology Panel (Genalyte Inc) is designed to detect antibodies to five SARS-CoV-2 antigens: nucleocapsid, Spike S1 RBD, Spike S1S2, Spike S2, and Spike S1 with in a multiplex format based on photonic ring resonance technology [72]. This system detects and measures with good reproducibility changes in resonance when antibodies bind to their respective antigens in the chip. The instrument automates the assay. Briefly, 10 µl of each serum sample were added to a sample well plate array containing required diluents and buffers. The plate and chip are loaded into the instrument. First, the chip is equilibrated with the diluent buffer to get baseline resonance. The serum sample is then charged over the chip to bind specific antibodies to antigens present on the chip. Next, chip is washed to remove low-affinity binders. Finally, specific antibodies of patients are detected with anti-IgG or -IgA or -IgM secondary antibodies.

#### Metabolomics analysis

Samples were prepared as previously described [73]. Briefly, serum samples were mixed with ice-cold extraction mixture (methanol/water, 9/1, v/v, with a mixture of internal standards), then centrifugated. Supernatants were collected for widely-targeted analysis of intracellular metabolites. *GC/MS analysis:* GC-MS/MS method was performed on a 7890B gas chromatography (Agilent Technologies, Waldbronn, Germany) coupled to a triple quadrupole 7000 C (Agilent Technologies, Waldbronn, Germany) equipped with a high sensitivity electronic impact source (EI) operating in positive mode. *Targeted analysis of bile acids:* Targeted analysis was performed on a RRLC 1260 system (Agilent Technologies, Waldbronn, Germany) coupled to a QTRAP 6500 + (Sciex) equipped with an electrospray source operating in negative mode. Gas temperature was set to 450 °C, with ion source gas 1 and 2 set to 30 and 70, respectively. *Targeted analysis of polyamines:* Targeted analysis was performed on a RRLC 1260 system (Agilent Technologies, Waldbronn, Germany) coupled to a QQQ 6410 (Agilent Technologies) equipped with an electrospray source operating in positive mode. The gas temperature was set to 350 °C with a gas flow of 12 l/min. The capillary voltage was set to 3.5 kV. *Targeted analysis of SCFA:* Targeted analysis was performed on a RRLC 1260 system (Agilent Technologies, Waldbronn, Germany) coupled to a QQQ 6410 (Agilent Technologies) equipped with an electrospray source operating in negative mode. Gas temperature was set to 350 °C with a gas flow of 12 L/min. The capillary voltage was set to 4.0 kV. *Pseudo-targeted analysis of intracellular metabolites:* The profiling experiment was performed with a Dionex Ultimate 3000 UHPLC system (Thermo Scientific) coupled to a Q-Exactive (Thermo Scientific) equipped with an electrospray source operating in both positive and negative mode and full scan mode from 100 to 1200 m/z. The Q-Exactive parameters were: sheath gas flow rate 55 au, auxiliary gas flow rate 15 au, spray voltage 3.3 kV, capillary temperature 300 °C, S-Lens RF level 55 V. The mass spectrometer was calibrated with sodium acetate solution dedicated to low mass calibration.

### Data analysis

#### Spectral flow cytometry

Fcs files were exported and analyzed using FlowJo software using the gating strategy showed in Supplementary Material, Fig. 2. Briefly, gates on CD45^+^, CD3^+^, or CD19^+^ from the myeloid, T cell and B panels, respectively, were exported in an fcs file. All exported gate**s** from one panel were used to generate an UMAP [74]. As shown on Supplementary Material Figs. 3 and 4, we used relative expression and manual gating strategy. For patients treated by anti-PD-1 monoclonal antibody, the gates including PD-1 were excluded of the analysis. For patients treated by anti-CD38 monoclonal antibody, the gates including CD38 were excluded of the analysis.

#### Representation of the results

Data representation was performed with software R v3.3.3 using tidyverse, dplyr, ggplot2, ggpubr, pheatmap, corrplot or Hmisc packages, or GraphPad Prism 7.

### Statistical analyses

Calculations and statistical tests were performed either with R v3.3.3 or Prism 7 (GraphPad, San Diego, CA, USA). Unless stated, *P* values are two-sided with 95% confidence intervals for the reported statistic of interest. Individual data points representing the measurement from one patient are systematically calculated from the corresponding distribution. Biological parameters associated to statistically significant differences between groups were considered for the data visualization described below. Group comparison was performed using one-way ANOVA with the lmer function of the lme4 R package. The p-values were computed with the Kenward–Roger method, available in the lmertest R package. Spearman correlations were computed using Hmisc and Pheatmap R package. Hierarchical clustering of the patient’s factors was performed using the hclust R package. The redundancy analysis (RDA) was performed using the vegan R package to explore the association between the clinical variables and the biological parameter correlation latent structure. The RDA performs variance decomposition such as principal component analysis, but including additional supervised components depending on the explanatory variables (e.g., clinical factors). The association of the clinical factors with the biological parameter correlation latent structure was tested using a permutation test. Kaplan–Meier methodology was used to estimate the probability of overall survival as well as to visualize the median time of SARS-CoV-2 RNA shedding for each group (HCW and Cancer). One-way ANOVA (paired and unpaired) with Kenward–Roger method was used to calculate *P* value between ALC among groups of viral RNA shedding and Ct values. Chi-Square, Fischer test were used `to calculate the differences in proportion between groups. Comparing two groups, Mann–Whitney test was used. Univariate analyses were performed with the Cox regression model. *P* < 0.05 was considered significant. Multivariate Cox analysis was performed using the survival R package stratified for the cohort and adjusted for the age, ECOG performance status, gender and metastatic status and hematological malignancy.

## Supplementary information

Supplementary material Figure 1

Supplementary material Figure 2,3,4

Supplementary material tables
