# Supplementary figures and images for: Prolonged SARS-CoV-2 RNA virus shedding and lymphopenia are hallmarks of COVID-19 in cancer patients with poor prognosis
Source: Cell Death Differ. 2021 Jul 6;28(12):3297–315. doi: 10.1038/s41418-021-00817-9 (PMC8259103; doi:10.1038/s41418-021-00817-9)

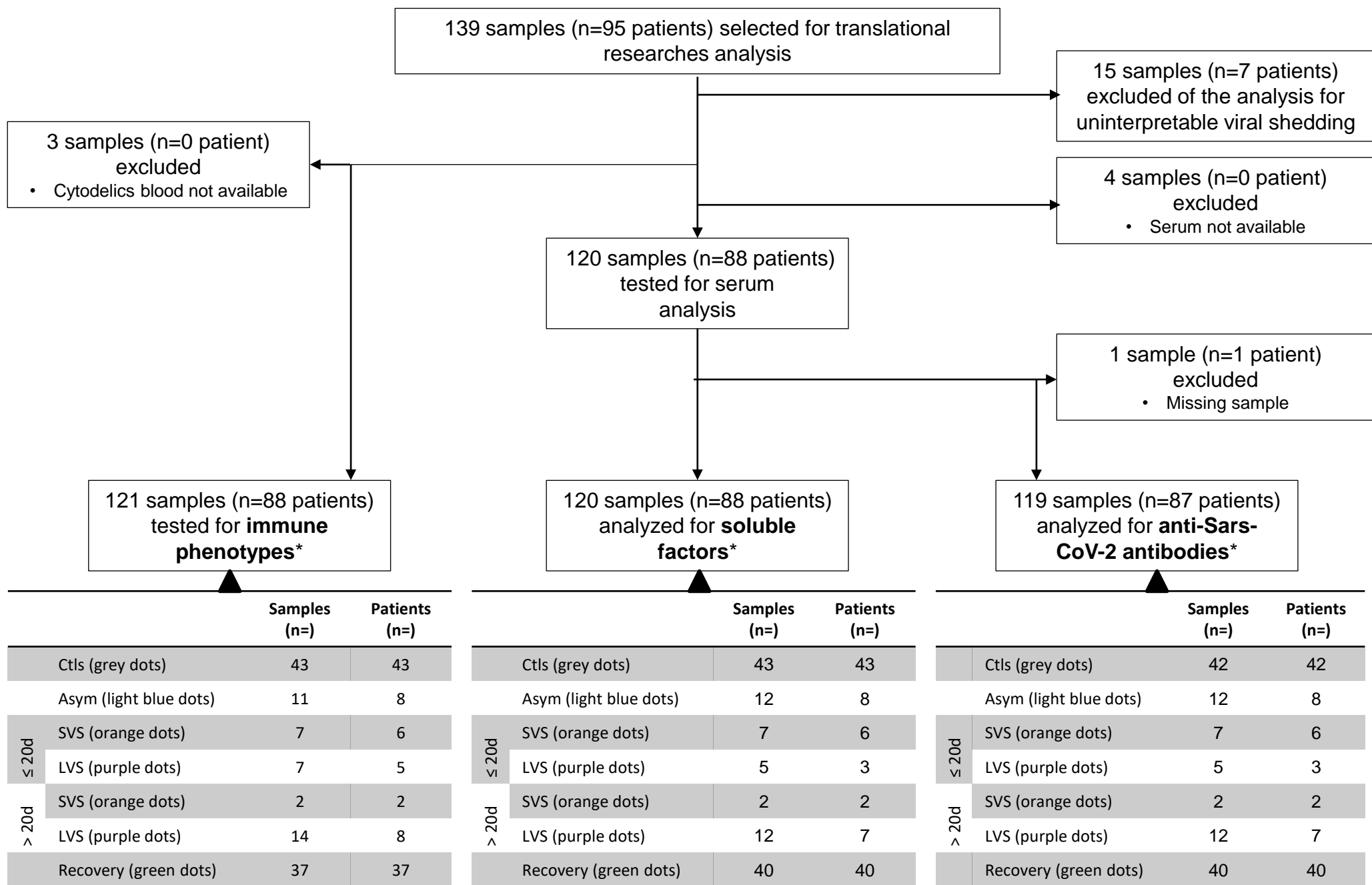

**Supplementary Material, Figure 1**

Supplement: Supplementary file 1 — Supplementary material Figure 1 [file 41418_2021_817_MOESM1_ESM.pdf]

**Example, 1 patient**

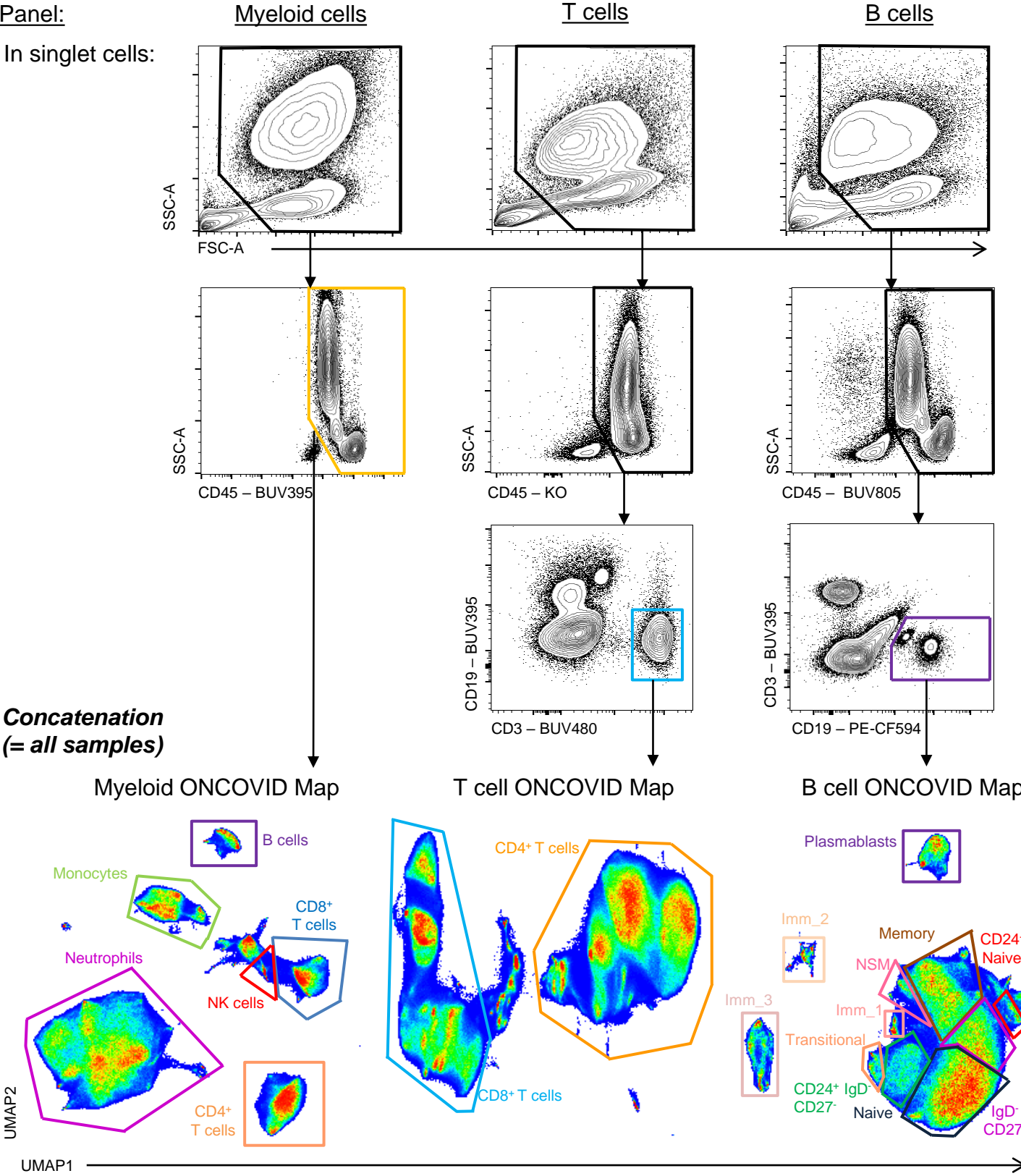

**Supplementary Material, Figure 2**

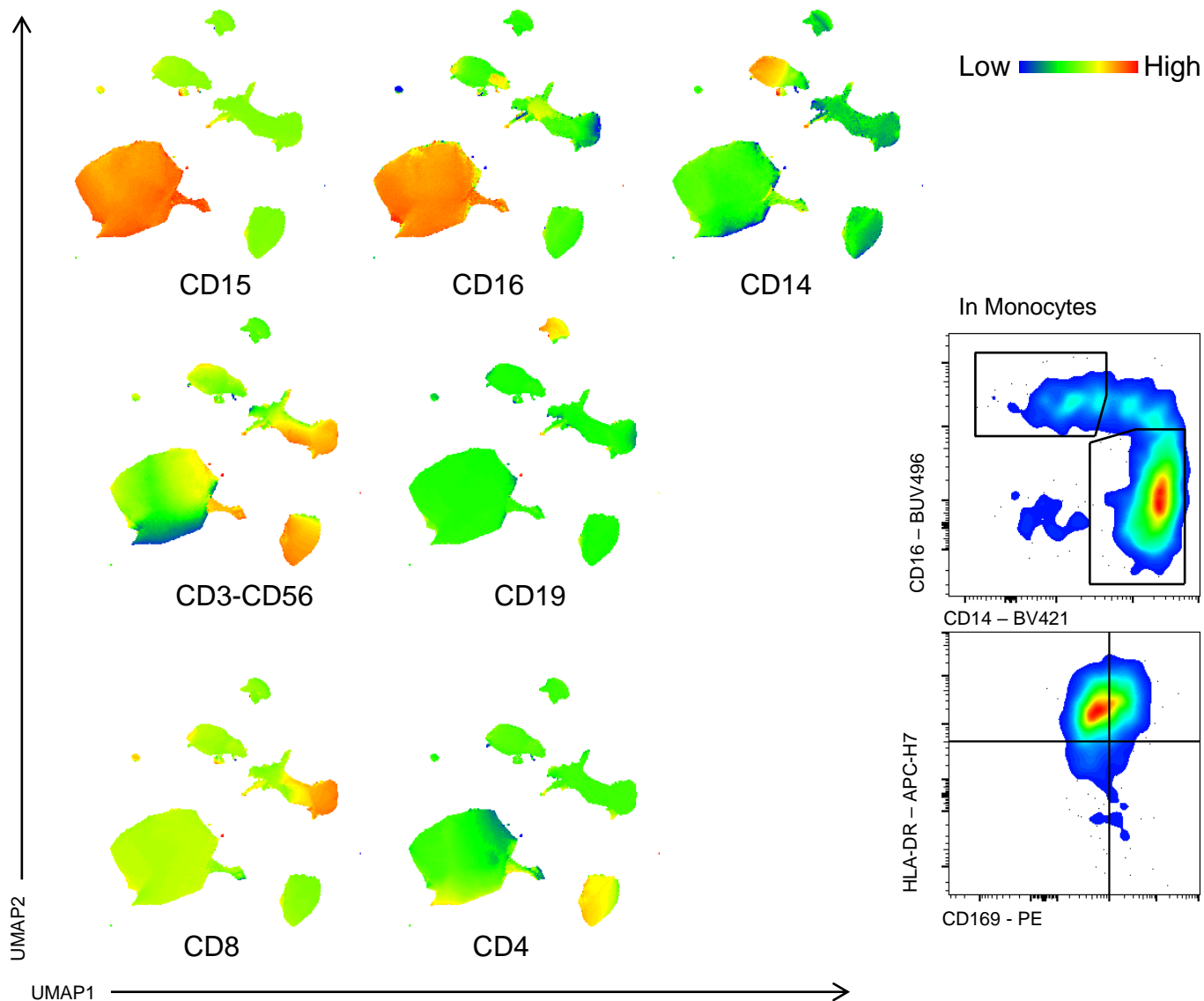

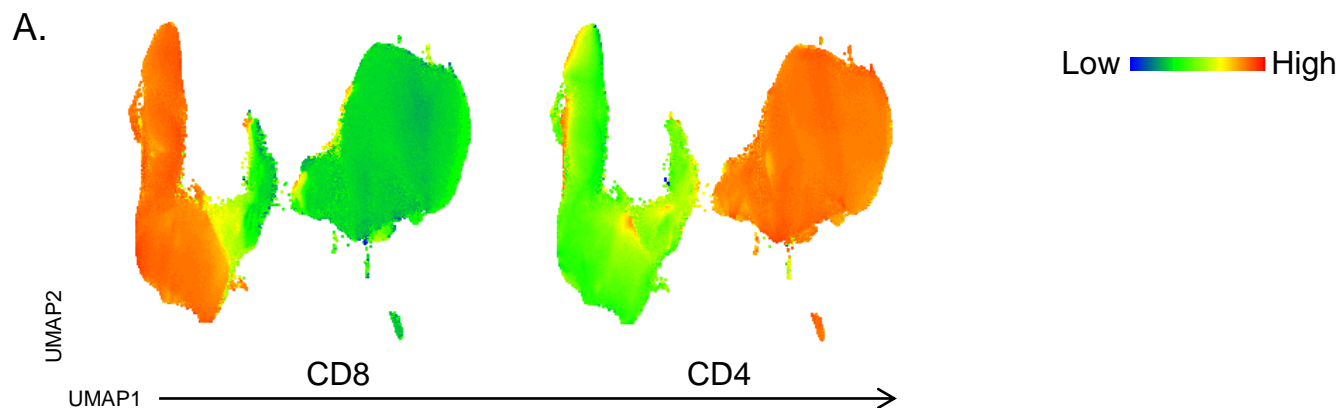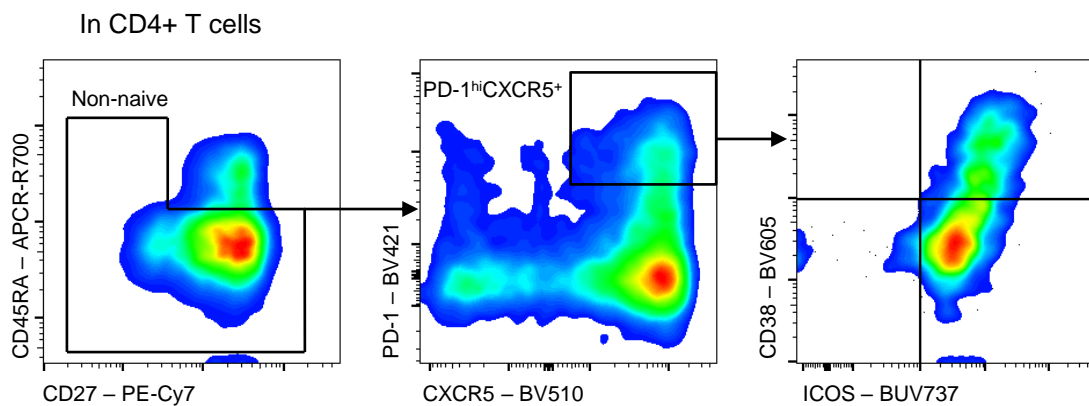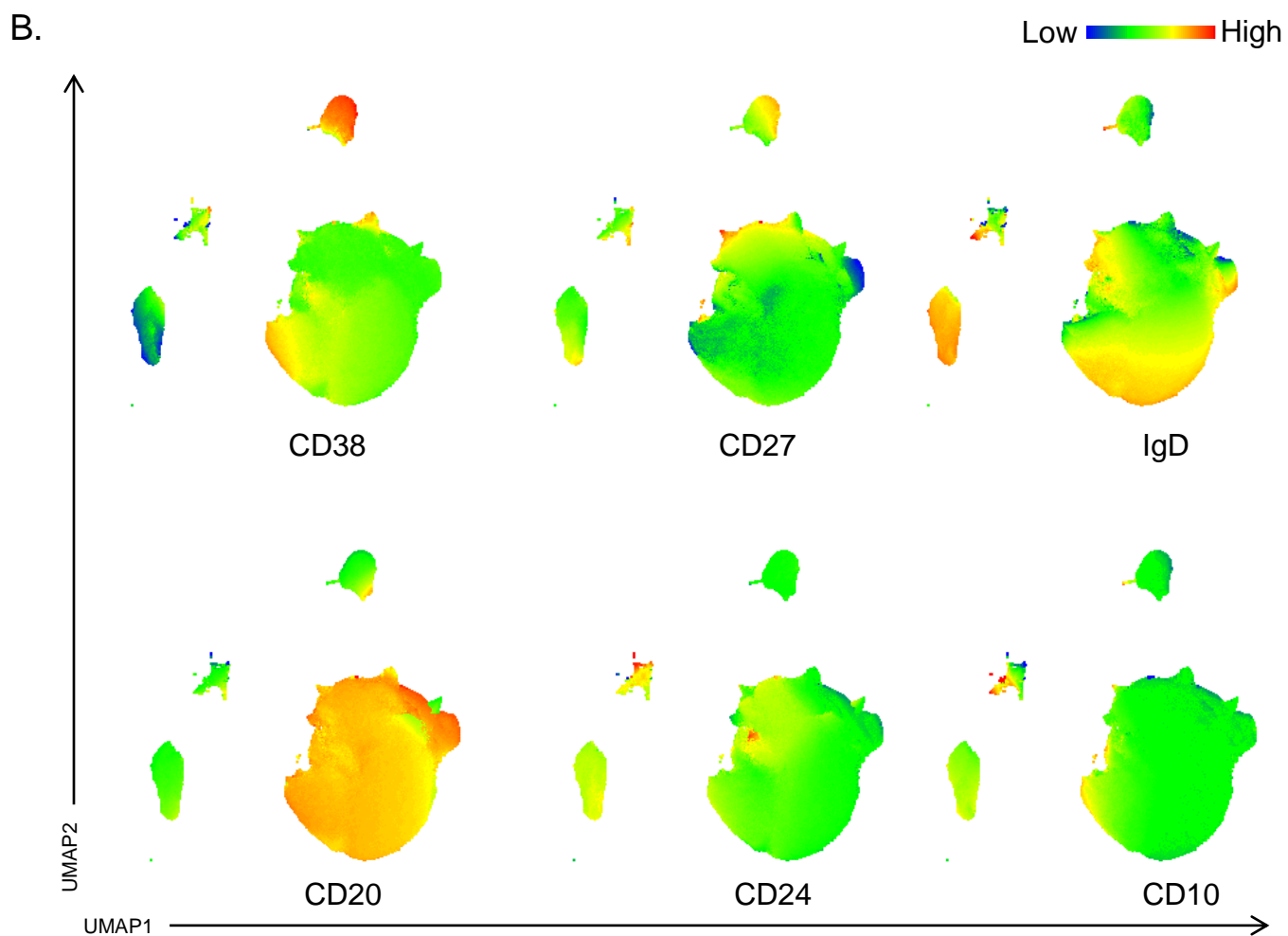

Supplement: Supplementary file 2 — Supplementary material Figure 2,3,4 [file 41418_2021_817_MOESM2_ESM.pdf]
