# Supplementary material for: Prolonged SARS-CoV-2 RNA virus shedding and lymphopenia are hallmarks of COVID-19 in cancer patients with poor prognosis
Source: Cell Death Differ. 2021 Jul 6;28(12):3297–315. doi: 10.1038/s41418-021-00817-9 (PMC8259103; doi:10.1038/s41418-021-00817-9)
Supplement: Supplementary file 3 — Supplementary material tables [file 41418_2021_817_MOESM3_ESM.pptx]

## Slide 1
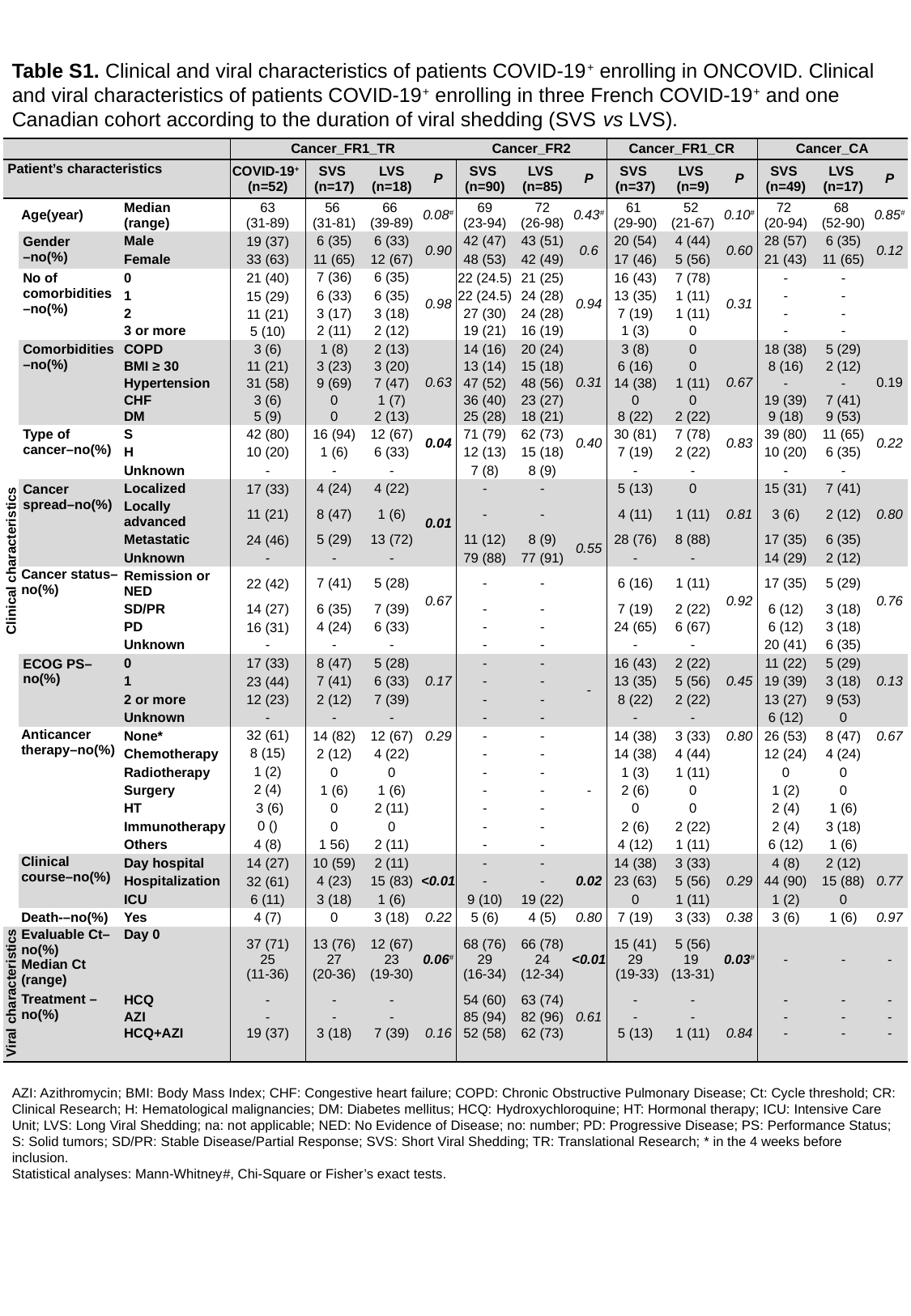

Table S1. Clinical and viral characteristics of patients COVID-19+ enrolling in ONCOVID. Clinical and viral characteristics of patients COVID-19+ enrolling in three French COVID-19+ and one Canadian cohort according to the duration of viral shedding (SVS vs LVS).
| | | | Cancer\_FR1\_TR | Cancer\_FR1\_TR | | | Cancer\_FR2 | | | Cancer\_FR1\_CR | | | Cancer\_CA | | |
| --- | --- | --- | --- | --- | --- | --- | --- | --- | --- | --- | --- | --- | --- | --- | --- |
| Patient’s characteristics | | | COVID-19+ (n=52) | SVS (n=17) | LVS (n=18) | P | SVS (n=90) | LVS (n=85) | P | SVS (n=37) | LVS (n=9) | P | SVS (n=49) | LVS (n=17) | P |
| Clinical characteristics | Age(year) | Median (range) | 63 (31-89) | 56 (31-81) | 66 (39-89) | 0.08# | 69 (23-94) | 72 (26-98) | 0.43# | 61 (29-90) | 52 (21-67) | 0.10# | 72 (20-94) | 68 (52-90) | 0.85# |
| | Gender –no(%) | Male | 19 (37) | 6 (35) | 6 (33) | 0.90 | 42 (47) | 43 (51) | 0.6 | 20 (54) | 4 (44) | 0.60 | 28 (57) | 6 (35) | 0.12 |
| | | Female | 33 (63) | 11 (65) | 12 (67) | | 48 (53) | 42 (49) | | 17 (46) | 5 (56) | | 21 (43) | 11 (65) | |
| | No of comorbidities–no(%) | 0 | 21 (40) | 7 (36) | 6 (35) | 0.98 | 22 (24.5) | 21 (25) | 0.94 | 16 (43) | 7 (78) | 0.31 | - | - | |
| | | 1 | 15 (29) | 6 (33) | 6 (35) | | 22 (24.5) | 24 (28) | 0.94 | 13 (35) | 1 (11) | | - | - | |
| | | 2 | 11 (21) | 3 (17) | 3 (18) | | 27 (30) | 24 (28) | | 7 (19) | 1 (11) | | - | - | |
| | | 3 or more | 5 (10) | 2 (11) | 2 (12) | | 19 (21) | 16 (19) | | 1 (3) | 0 | | - | - | |
| | Comorbidities–no(%) | COPD | 3 (6) | 1 (8) | 2 (13) | 0.63 | 14 (16) | 20 (24) | 0.31 | 3 (8) | 0 | 0.67 | 18 (38) | 5 (29) | 0.19 |
| | | BMI ≥ 30 | 11 (21) | 3 (23) | 3 (20) | | 13 (14) | 15 (18) | | 6 (16) | 0 | | 8 (16) | 2 (12) | |
| | | Hypertension | 31 (58) | 9 (69) | 7 (47) | | | | | | | | | | |
| | | | | | | | | | | | | | - | - | |
| | | | | | | | 47 (52) | 48 (56) | | 14 (38) | 1 (11) | | | | |
| | | CHF | 3 (6) | 0 | 1 (7) | | 36 (40) | 23 (27) | | 0 | 0 | | 19 (39) | 7 (41) | |
| | | DM | 5 (9) | 0 | 2 (13) | | | | | | | | | | |
| | | | | | | | 25 (28) | 18 (21) | | 8 (22) | 2 (22) | | | | |
| | | | | | | | | | | | | | 9 (18) | 9 (53) | |
| | Type of cancer–no(%) | S | 42 (80) | 16 (94) | 12 (67) | 0.04 | 71 (79) | 62 (73) | 0.40 | 30 (81) | 7 (78) | 0.83 | 39 (80) | 11 (65) | 0.22 |
| | | H | 10 (20) | 1 (6) | 6 (33) | | 12 (13) | 15 (18) | | 7 (19) | 2 (22) | | 10 (20) | 6 (35) | |
| | | Unknown | - | - | - | | 7 (8) | 8 (9) | | - | - | | - | - | |
| | Cancer spread–no(%) | Localized | 17 (33) | 4 (24) | 4 (22) | 0.01 | - | - | | 5 (13) | 0 | 0.81 | 15 (31) | 7 (41) | 0.80 |
| | | Locally advanced | 11 (21) | 8 (47) | 1 (6) | | - | - | | 4 (11) | 1 (11) | | 3 (6) | 2 (12) | |
| | | Metastatic | 24 (46) | 5 (29) | 13 (72) | | 11 (12) | 8 (9) | 0.55 | 28 (76) | 8 (88) | | 17 (35) | 6 (35) | |
| | | Unknown | - | - | - | | 79 (88) | 77 (91) | | - | - | | 14 (29) | 2 (12) | |
| | Cancer status–no(%) | Remission or NED | 22 (42) | 7 (41) | 5 (28) | 0.67 | - | - | | 6 (16) | 1 (11) | 0.92 | 17 (35) | 5 (29) | 0.76 |
| | | SD/PR | 14 (27) | 6 (35) | 7 (39) | | - | - | | 7 (19) | 2 (22) | | 6 (12) | 3 (18) | |
| | | PD | 16 (31) | 4 (24) | 6 (33) | | - | - | | 24 (65) | 6 (67) | | 6 (12) | 3 (18) | |
| | | Unknown | - | - | - | | - | - | | - | - | | 20 (41) | 6 (35) | |
| | ECOG PS–no(%) | 0 | 17 (33) | 8 (47) | 5 (28) | 0.17 | - | - | - | 16 (43) | 2 (22) | 0.45 | 11 (22) | 5 (29) | 0.13 |
| | | 1 | 23 (44) | 7 (41) | 6 (33) | | - | - | | 13 (35) | 5 (56) | | 19 (39) | 3 (18) | |
| | | 2 or more | 12 (23) | 2 (12) | 7 (39) | | - | - | | 8 (22) | 2 (22) | | 13 (27) | 9 (53) | |
| | | Unknown | - | - | - | | - | - | | - | - | | 6 (12) | 0 | |
| | Anticancer therapy–no(%) | None\* | 32 (61) | 14 (82) | 12 (67) | 0.29 | - | - | - | 14 (38) | 3 (33) | 0.80 | 26 (53) | 8 (47) | 0.67 |
| | | Chemotherapy | 8 (15) | 2 (12) | 4 (22) | | - | - | | 14 (38) | 4 (44) | | 12 (24) | 4 (24) | |
| | | Radiotherapy | 1 (2) | 0 | 0 | | - | - | | 1 (3) | 1 (11) | | 0 | 0 | |
| | | Surgery | 2 (4) | 1 (6) | 1 (6) | | - | - | | 2 (6) | 0 | | 1 (2) | 0 | |
| | | HT | 3 (6) | 0 | 2 (11) | | - | - | | 0 | 0 | | 2 (4) | 1 (6) | |
| | | Immunotherapy | 0 () | 0 | 0 | | - | - | | 2 (6) | 2 (22) | | 2 (4) | 3 (18) | |
| | | Others | 4 (8) | 1 56) | 2 (11) | | - | - | | 4 (12) | 1 (11) | | 6 (12) | 1 (6) | |
| | Clinical course–no(%) | Day hospital | 14 (27) | 10 (59) | 2 (11) | <0.01 | - | - | 0.02 | 14 (38) | 3 (33) | 0.29 | 4 (8) | 2 (12) | 0.77 |
| | | Hospitalization | 32 (61) | 4 (23) | 15 (83) | | - | - | | 23 (63) | 5 (56) | | 44 (90) | 15 (88) | |
| | | ICU | 6 (11) | 3 (18) | 1 (6) | | 9 (10) | 19 (22) | | 0 | 1 (11) | | 1 (2) | 0 | |
| | Death-–no(%) | Yes | 4 (7) | 0 | 3 (18) | 0.22 | 5 (6) | 4 (5) | 0.80 | 7 (19) | 3 (33) | 0.38 | 3 (6) | 1 (6) | 0.97 |
| Viral characteristics | Evaluable Ct–no(%) Median Ct (range) | Day 0 | 37 (71) 25 (11-36) | 13 (76) 27 (20-36) | 12 (67) 23 (19-30) | 0.06# | 68 (76) 29 (16-34) | 66 (78) 24 (12-34) | <0.01 | 15 (41) 29 (19-33) | 5 (56) 19 (13-31) | 0.03# | - | - | - |
| | Treatment –no(%) | HCQ | - | - | - | | 54 (60) | 63 (74) | 0.61 | - | - | | - | - | - |
| | | AZI | - | - | - | | 85 (94) | 82 (96) | | - | - | | - | - | - |
| | | HCQ+AZI | 19 (37) | 3 (18) | 7 (39) | 0.16 | 52 (58) | 62 (73) | | 5 (13) | 1 (11) | 0.84 | - | - | - |
| | | | | | | | | | | | | | | | |
AZI: Azithromycin; BMI: Body Mass Index; CHF: Congestive heart failure; COPD: Chronic Obstructive Pulmonary Disease; Ct: Cycle threshold; CR: Clinical Research; H: Hematological malignancies; DM: Diabetes mellitus; HCQ: Hydroxychloroquine; HT: Hormonal therapy; ICU: Intensive Care Unit; LVS: Long Viral Shedding; na: not applicable; NED: No Evidence of Disease; no: number; PD: Progressive Disease; PS: Performance Status; S: Solid tumors; SD/PR: Stable Disease/Partial Response; SVS: Short Viral Shedding; TR: Translational Research; * in the 4 weeks before inclusion.
Statistical analyses: Mann-Whitney#, Chi-Square or Fisher’s exact tests.

## Slide 2
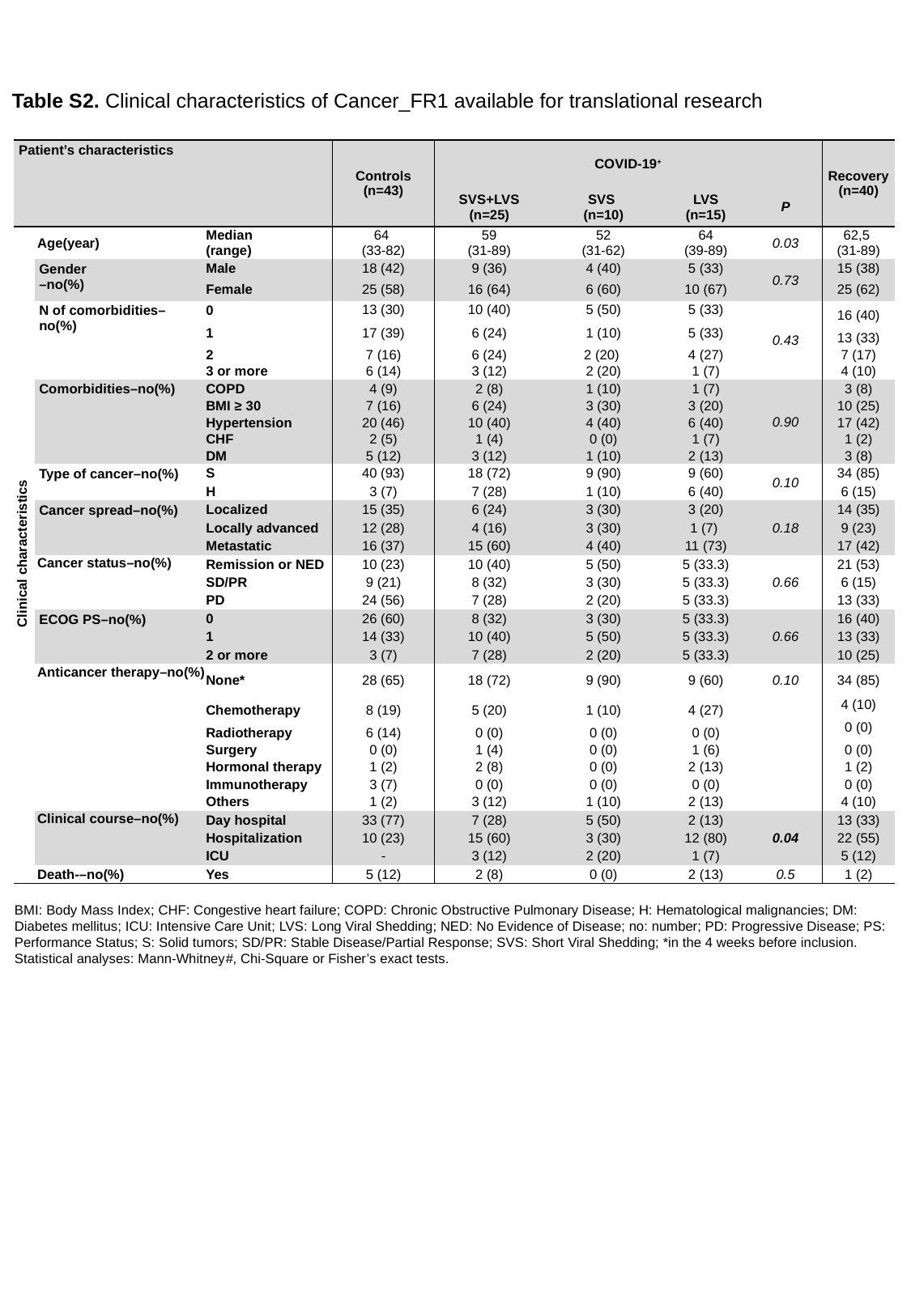

Table S2. Clinical characteristics of Cancer_FR1 available for translational research
| Patient’s characteristics | | | Controls (n=43) | COVID-19+ | COVID+ | | | Recovery (n=40) |
| --- | --- | --- | --- | --- | --- | --- | --- | --- |
| | | | | SVS+LVS (n=25) | SVS (n=10) | LVS (n=15) | P | |
| Clinical characteristics | Age(year) | Median (range) | 64 (33-82) | 59 (31-89) | 52 (31-62) | 64 (39-89) | 0.03 | 62,5 (31-89) |
| | Gender –no(%) | Male | 18 (42) | 9 (36) | 4 (40) | 5 (33) | 0.73 | 15 (38) |
| | | Female | 25 (58) | 16 (64) | 6 (60) | 10 (67) | | 25 (62) |
| | N of comorbidities–no(%) | 0 | 13 (30) | 10 (40) | 5 (50) | 5 (33) | 0.43 | 16 (40) |
| | | 1 | 17 (39) | 6 (24) | 1 (10) | 5 (33) | | |
| | | | | | | | | 13 (33) |
| | | 2 | 7 (16) | 6 (24) | 2 (20) | 4 (27) | | 7 (17) |
| | | 3 or more | 6 (14) | 3 (12) | 2 (20) | 1 (7) | | 4 (10) |
| | Comorbidities–no(%) | COPD | 4 (9) | 2 (8) | 1 (10) | 1 (7) | 0.90 | 3 (8) |
| | | BMI ≥ 30 | 7 (16) | 6 (24) | 3 (30) | 3 (20) | | 10 (25) |
| | | Hypertension | 20 (46) | 10 (40) | 4 (40) | 6 (40) | | 17 (42) |
| | | CHF | 2 (5) | 1 (4) | 0 (0) | 1 (7) | | 1 (2) |
| | | DM | 5 (12) | 3 (12) | 1 (10) | 2 (13) | | 3 (8) |
| | Type of cancer–no(%) | S | 40 (93) | 18 (72) | 9 (90) | 9 (60) | 0.10 | 34 (85) |
| | | H | 3 (7) | 7 (28) | 1 (10) | 6 (40) | | 6 (15) |
| | Cancer spread–no(%) | Localized | 15 (35) | 6 (24) | 3 (30) | 3 (20) | 0.18 | 14 (35) |
| | | Locally advanced | 12 (28) | 4 (16) | 3 (30) | 1 (7) | | 9 (23) |
| | | Metastatic | 16 (37) | 15 (60) | 4 (40) | 11 (73) | | 17 (42) |
| | Cancer status–no(%) | Remission or NED | 10 (23) | 10 (40) | 5 (50) | 5 (33.3) | 0.66 | 21 (53) |
| | | SD/PR | 9 (21) | 8 (32) | 3 (30) | 5 (33.3) | | 6 (15) |
| | | PD | 24 (56) | 7 (28) | 2 (20) | 5 (33.3) | | 13 (33) |
| | ECOG PS–no(%) | 0 | 26 (60) | 8 (32) | 3 (30) | 5 (33.3) | 0.66 | 16 (40) |
| | | 1 | 14 (33) | 10 (40) | 5 (50) | 5 (33.3) | | 13 (33) |
| | | 2 or more | 3 (7) | 7 (28) | 2 (20) | 5 (33.3) | | 10 (25) |
| | Anticancer therapy–no(%) | None\* | 28 (65) | 18 (72) | 9 (90) | 9 (60) | 0.10 | 34 (85) |
| | | Chemotherapy | 8 (19) | 5 (20) | 1 (10) | 4 (27) | | |
| | | | | | | | | 4 (10) |
| | | | | | | | 1 | 0 (0) |
| | | Radiotherapy | 6 (14) | 0 (0) | 0 (0) | 0 (0) | | |
| | | Surgery | 0 (0) | 1 (4) | 0 (0) | 1 (6) | | 0 (0) |
| | | Hormonal therapy | 1 (2) | 2 (8) | 0 (0) | 2 (13) | | 1 (2) |
| | | Immunotherapy | 3 (7) | 0 (0) | 0 (0) | 0 (0) | | 0 (0) |
| | | Others | 1 (2) | 3 (12) | 1 (10) | 2 (13) | | 4 (10) |
| | Clinical course–no(%) | Day hospital | 33 (77) | 7 (28) | 5 (50) | 2 (13) | 0.04 | 13 (33) |
| | | Hospitalization | 10 (23) | 15 (60) | 3 (30) | 12 (80) | | 22 (55) |
| | | ICU | - | 3 (12) | 2 (20) | 1 (7) | | 5 (12) |
| | Death-–no(%) | Yes | 5 (12) | 2 (8) | 0 (0) | 2 (13) | 0.5 | 1 (2) |
BMI: Body Mass Index; CHF: Congestive heart failure; COPD: Chronic Obstructive Pulmonary Disease; H: Hematological malignancies; DM: Diabetes mellitus; ICU: Intensive Care Unit; LVS: Long Viral Shedding; NED: No Evidence of Disease; no: number; PD: Progressive Disease; PS: Performance Status; S: Solid tumors; SD/PR: Stable Disease/Partial Response; SVS: Short Viral Shedding; *in the 4 weeks before inclusion.
Statistical analyses: Mann-Whitney#, Chi-Square or Fisher’s exact tests.
